# Genomic adaptation of *Pseudomonas* strains to acidity and antibiotics in hydrothermal vents at Kolumbo submarine volcano, Greece

**DOI:** 10.1038/s41598-020-79359-y

**Published:** 2021-01-14

**Authors:** Panos Bravakos, Manolis Mandalakis, Paraskevi Nomikou, Thekla I. Anastasiou, Jon Bent Kristoffersen, Melanthia Stavroulaki, Stephanos Kilias, Georgios Kotoulas, Antonios Magoulas, Paraskevi N. Polymenakou

**Affiliations:** 1 Institute of Marine Biology, Biotechnology and Aquaculture, Hellenic Centre for Marine Research (IMBBC-HCMR), Gournes Pediados, Heraklion Crete, Greece; 2 Department of Geology and Geoenvironment, National and Kapodistrian University of Athens, Athens, Greece

**Keywords:** Environmental impact, Marine biology

## Abstract

Although the rise of antibiotic and multidrug resistant bacteria is one of the biggest current threats to human health, our understanding of the mechanisms involved in antibiotic resistance selection remains scarce. We performed whole genome sequencing of 21 *Pseudomonas* strains, previously isolated from an active submarine volcano of Greece, the Kolumbo volcano. Our goal was to identify the genetic basis of the enhanced co-tolerance to antibiotics and acidity of these *Pseudomonas* strains. Pangenome analysis identified 10,908 Gene Clusters (GCs). It revealed that the numbers of phage-related GCs and sigma factors, which both provide the mechanisms of adaptation to environmental stressors, were much higher in the high tolerant *Pseudomonas* strains compared to the rest ones. All identified GCs of these strains were associated with antimicrobial and multidrug resistance. The present study provides strong evidence that the CO_2_-rich seawater of the volcano associated with low pH might be a reservoir of microorganisms carrying multidrug efflux-mediated systems and pumps. We, therefore, suggest further studies of other extreme environments (or ecosystems) and their associated physicochemical parameters (or factors) in the rise of antibiotic resistance.

## Introduction

During the last decade, there is a growing global concern about the rise of antibiotic and multidrug resistant bacteria resulting from the pressure of antibiotic usage^1,2^. Previous research on antibiotic resistance (AR) was limited in clinical environments, but due to an increase in life-threatening infections, research on AR in natural habitats emerged^3,4^. Recent studies on natural environments have revealed vast genetic reservoirs of AR genes^5,6^. These include soils^6,7^, glaciers^8^, seawater and penguin fecal samples^9^ and animals^10^. In 2015, Hatosy and Martiny^11^ uncovered a previously unknown diversity of AR genes among marine environments and suggested the ocean as a global reservoir of clinically relevant and potential novel AR genes.

Recent environmental studies have shown that the increased AR resistance of bacteria in natural habitats, can be associated with environmental stressors such as the pH reduction. The first observation was made in 2009 by Vega Thurber et al.^12^, who noticed that stressors, such as the pH decrease, can increase the abundance of AR genes in coral-associated bacterial communities^3,12^. In 2011, Meron et al.^13^ recorded an increase in isolated bacteria producing antibacterial activity from corals maintained at pH 7.3 as compared with isolated strains obtained from corals maintained at pH 8.2. Such observations raise special concerns as there is an alarming decline in global surface ocean pH, (also known as ocean acidification^14^), which can potentially trigger an increase in antimicrobial activity and the selection of antibiotic resistance genes.

A good candidate microorganism to study the bacterial evolution in a range of environmental stressors, such as the pH decline, and its role on the rise of antibiotic resistance, is the gram-negative cosmopolitan *Pseudomonas*, one of the most diverse bacterial genera within *Gammaproteobacteria*^15^*. Pseudomonas* members show an impressive metabolic and physiologic versatility^16–18^, and they have adopted mechanisms to promote their survival and persistence in various environments^19^.

*Pseudomonas* members are widely distributed in a submarine arc-volcano of the Hellenic volcanic arc, the Kolumbo volcano, which is characterized by natural CO_2_ efflux from seafloor hydrothermal vents^20,21^. This volcano, located 7 km northeast of Santorini island^22,23^, is a completely enclosed crater with steep vertical inner slopes and a flat floor at 500 m depth that is riddled with hydrothermal vents^22,24^. The bowl-shaped morphology of Kolumbo impedes vertical mixing. It, therefore, leads to the establishment of a dense, CO_2_-rich, acidic (as low as pH 5.0) seawater column extending for ~ 15 m above the crater floor vents^21,25^. Its seafloor hydrothermal vent field consists of active and inactive sulfide vent chimneys of spire or mound shape^26^. A variety of microbial mats of highly diverse microbial communities are covering the chimney/mound walls^20^, whereas the crater floor is covered with a thick layer of sediment consisting of highly complex microbial mats^27^.

In a recent study, a series of *Pseudomonas* strains isolated from the water column of the Kolumbo crater, were used to investigate whether the long-standing acidic conditions near the crater floor compared to the normal conditions of the surface seawater, may have an impact on the phenotypic traits of marine bacteria^4^. *Pseudomonas* strains isolated from the active area of Kolumbo volcano showed an enhanced co-tolerance to acidity and antibiotics. It was further suggested that an ocean’s pH decrease over the coming decades may favor the overall increase of *Pseudomonas* tolerance to antibiotics. A genome sequence analysis of these *Pseudomonas* isolates could further provide valuable insights into the genetic basis of this adaptation. With this in mind, we undertook genome sequencing for 21 of these strains that have been previously isolated from Kolumbo volcano, in order to identify the genetic features that shape the adaptability of the *Pseudomonas* members to the dense stratified acidic seawater column above the Kolumbo vents and to get a better understanding of the factors that influence antibiotic resistance.

## Results

### Genome analysis

**Figure 1:**
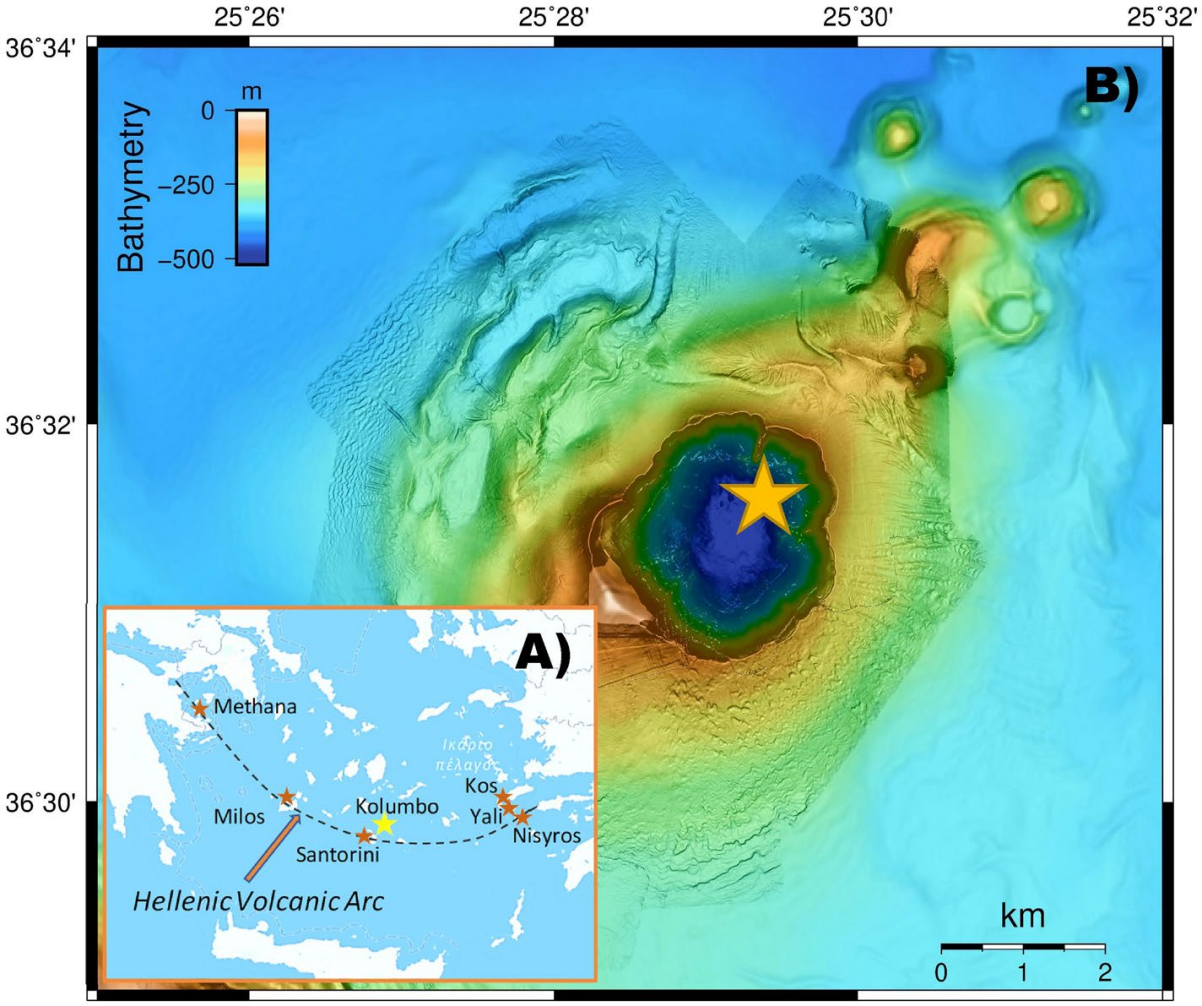
Maps and bottom topography of the sampling location. (A) Map showing the location of the Hellenic Volcanic Arc in the Aegean Sea and the study area of Kolumbo volcano. (B) High resolution Autonomous Underwater Vehicle (AUV)-collected bathymetry at 2 m resolution of Kolumbo crater showing the location of the surface and deep seawater samples that were used for *Pseudomonas* strains isolation. AUV data were collected during POS510 cruise, in 7 missions of AUV Abyss (GEOMAR), under the framework of the collaborative project “ANYDROS: Rifting and Hydrothermal Activity in the Cyclades Back-arc Basin” (modified from Nomikou et al.^70^). For the swath data processing, visualization, and bathymetric maps we used the freely available software packages MBsystem v.5.7.6 (https://www.mbari.org/products/research-software/mb-system/) and QGIS v.3.16 (https://www.qgis.org/en/site/).

**Figure 2:**
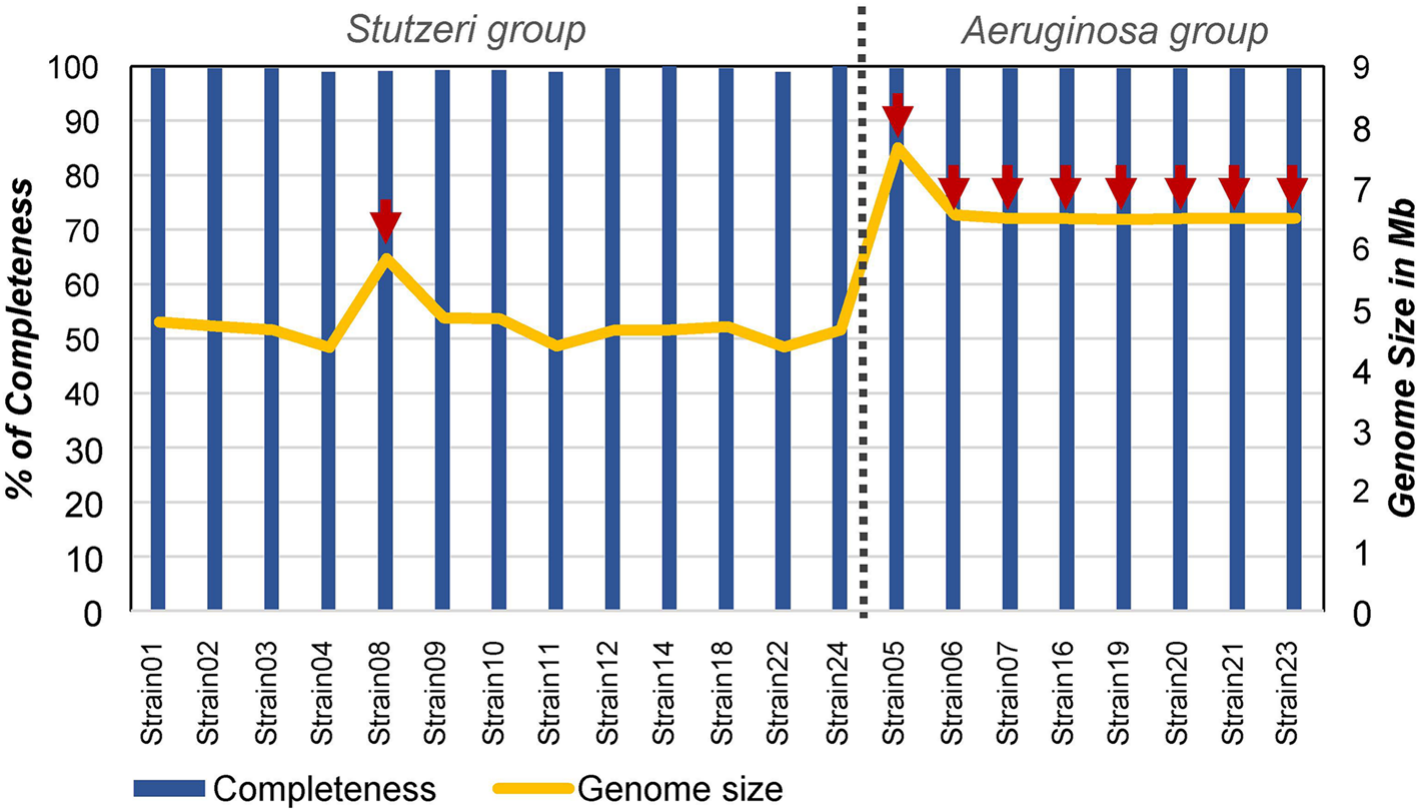
Statistics of whole genome sequencing analysis. Bars represent percentages of completeness per strain from CheckM analysis, while the line represents the genome size in Mb. Red arrows indicate the strains of high tolerance to acidity, antibiotics and heavy metals.

**Figure 3:**
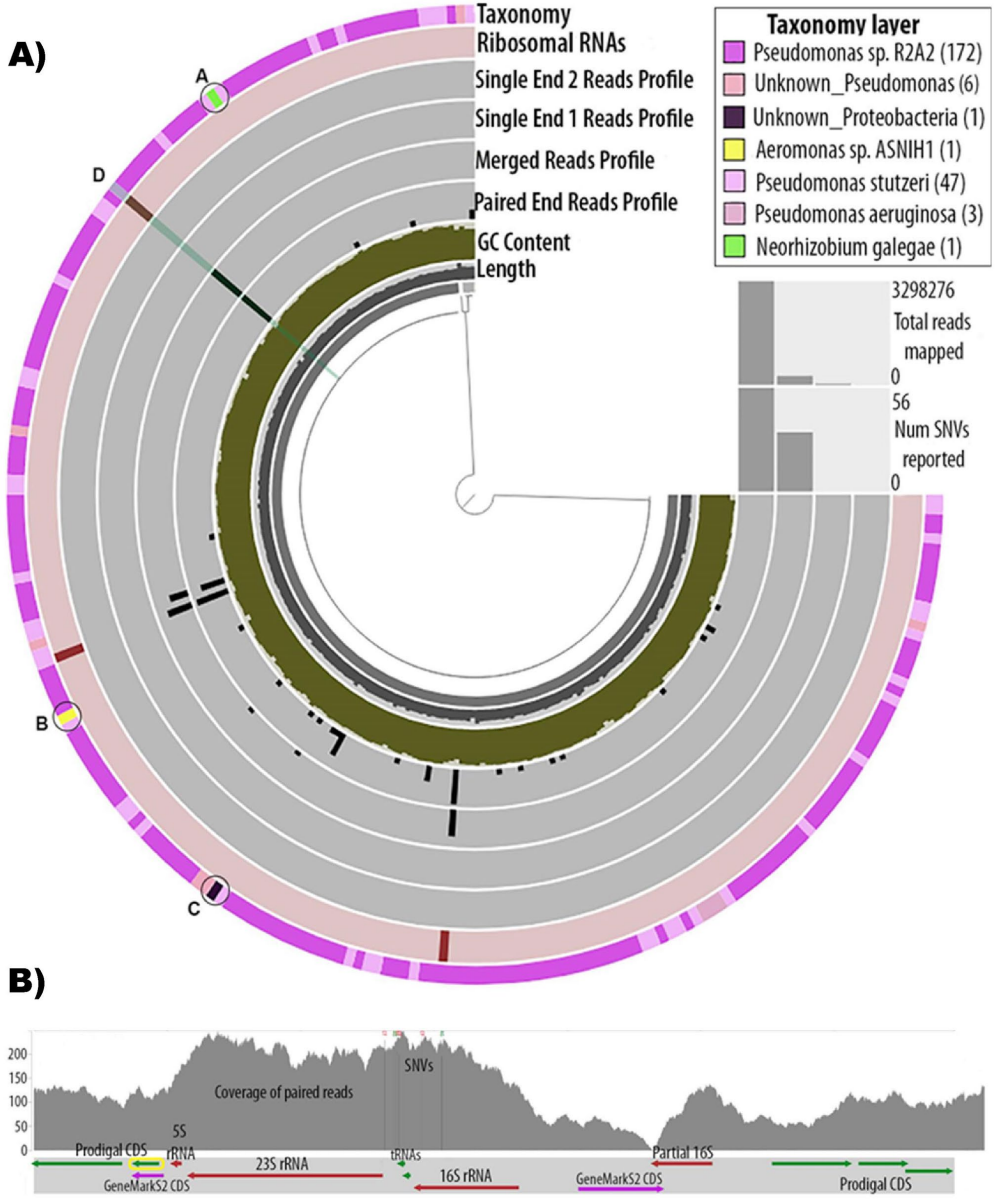
Genome analysis of a representative strain. The same analysis was applied for all strains. (A) Anvi’o representation of the genome assembly of Strain03. The tree structure in the inner circle represents the scaffolds of the genome. The black first inner layer is the length of the split for which all above layers are calculated, the dark-greened second layer is the GC content, the third, fourth, fifth and sixth layers represent the variability (SNVs) (in black) found in the paired end reads, merged reads, single end from pair 1 reads and single end from pair 2 reads, respectively. The seventh layer represents the rRNAs found in the genome, and the last outer layer represents the gene-level taxonomy. Coloration of the outer layer is according to taxonomy assignment as indicated in the upper right insert. Anvi’o splits that were not taxonomically assigned under the *Pseudomonaceae* family are marked as A, B and C. The highlighted split D, is explored further in Fig. 5. (B) Detailed view of a part of the genome of Strain03. In the upper frame the coverage of the paired end reads is depicted in the gray graph. Vertical lines with colored nucleotide letters on top depict position of the SNVs. The lower frame shows the genes found in the particular split, their length and orientation. An rRNA operon (16S rRNA, 23S rRNA and 5S rRNA) is shown in this split. A partial 16S rRNA with only 55% of its sequence aligned to 16S rRNA is also shown to be found outside of the rRNA operon. Most CDS shown were predicted by Prodigal (green arrows), except for the two CDS predicted by GeneMarkS2 (purple arrows), as indicated in the figure. The annotation of the yellow framed CDS is given in Table 2.

A total of 21 strains were chosen based on previously published data to represent both surface (5–90 m) and deep (430 m and 495 m) seawater layers of the Kolumbo volcano and their susceptibility to various environmental stressors including acidity, six commonly used antibiotics and heavy metals^4^ (Fig. 1; Table 1; Supplementary Table S1). More specifically, 9 of the strains stemmed from the surface, and the remaining 12 strains originated from the deep seawater layers. All surface seawater strains and three deep seawater strains showed low acid, antibiotic and heavy metals tolerance, whereas the remaining nine strains from the deep seawater showed high tolerance to the investigated environmental stressors^4^ (Table 1, Supplementary Table S1). Genome sequencing analysis of all strains with MiSeq technology and subsequent assembly with Spades^28^ allowed to obtain draft assemblies for all isolates. These assemblies were inspected for contamination from other strains and corrected, further extended using complete genomes from related *Pseudomonas* strains, and gaps were filled by mapping the raw sequence data against these assemblies. The final assemblies contained between 4 and 52 scaffolds for each genome, and genome size varied from 4.3 Mb to 6.4 Mb (Fig. 2). Single Nucleotide Variants (SNVs) were found in few positions across the genomes (Fig. 3). CheckM analysis^29^ showed completeness equal to or higher than 98.9% for all genomes (Fig. 2), whereas Busco analysis^30^ showed completeness equal to or higher than 99.7% (Supplementary Table S2). Functional annotations using a total of 28 databases covered more than 97% of the Coding Sequences (CDS), which were predicted by Prodigal v.2.6^31^. Details are provided in Supplementary Data.

**Table 1:** Sampling depth, taxonomy assignment and phenotypic traits (i.e. high or low tolerance to acidity, antibiotics and heavy metals) of the 21 strains.

Strain code	Depth (m)	Closest culture representative	ANI (%)	Main groups	Subgroups
**Low tolerance**					
Strain01	45	*Pseudomonas* sp. R2A2	> 97.1	*Stutzeri *	*Stutzeri 1*
Strain02	90	*Pseudomonas* sp. R2A2	> 97.1	*Stutzeri *	*Stutzeri 1*
**Strain03**	5	*Pseudomonas* sp. R2A2	> 97.1	*Stutzeri *	*Stutzeri 1*
Strain04	495	*P. balearica* DSM_6083	> 98.51	*Stutzeri *	*Balearica*
Strain09	20	*Pseudomonas* sp. MT-1	> 97.1	*Stutzeri *	*Xanthomarina 1*
Strain10	20	*Pseudomonas* sp. MT-1	> 97.1	*Stutzeri *	*Xanthomarina 1*
**Strain11**	20	*P. balearica* DSM_6083	> 98.51	*Stutzeri *	*Balearica*
Strain12	20	*Pseudomonas* sp. R2A2	> 97.1	*Stutzeri *	*Stutzeri 1*
**Strain14**	90	*P. stutzeri* 1W1-1A	> 97.3	*Stutzeri *	*Stutzeri 2*
Strain18	90	*Pseudomonas* sp. R2A2	> 97.1	*Stutzeri *	*Stutzeri 1*
Strain22	495	*P. balearica* DSM_6083	> 98.51	*Stutzeri *	*Balearica*
Strain24	495	*P. stutzeri* 1W1-1A	> 97.3	*Stutzeri *	*Stutzeri 2*
**High tolerance**					
Strain05	495	*P. aeruginosa* MTB-1	> 98.97	*Aeruginosa*	*Aeruginosa*
Strain06	495	*P. aeruginosa* MTB-1	> 98.97	*Aeruginosa*	*Aeruginosa*
Strain07	495	*P. aeruginosa* MTB-1	> 98.97	*Aeruginosa*	*Aeruginosa*
**Strain08**	430	*P. xanthomarina* LMG 23572	88.8	*Stutzeri *	*Xanthomarina 2*
Strain16	495	*P. aeruginosa* MTB-1	> 98.97	*Aeruginosa*	*Aeruginosa*
Strain19	495	*P. aeruginosa* MTB-1	> 98.97	*Aeruginosa*	*Aeruginosa*
Strain20	495	*P. aeruginosa* MTB-1	> 98.97	*Aeruginosa*	*Aeruginosa*
Strain21	495	*P. aeruginosa* MTB-1	> 98.97	*Aeruginosa*	*Aeruginosa*
**Strain23**	495	*P. aeruginosa* MTB-1	> 98.97	*Aeruginosa*	*Aeruginosa*

**Figure 4:**
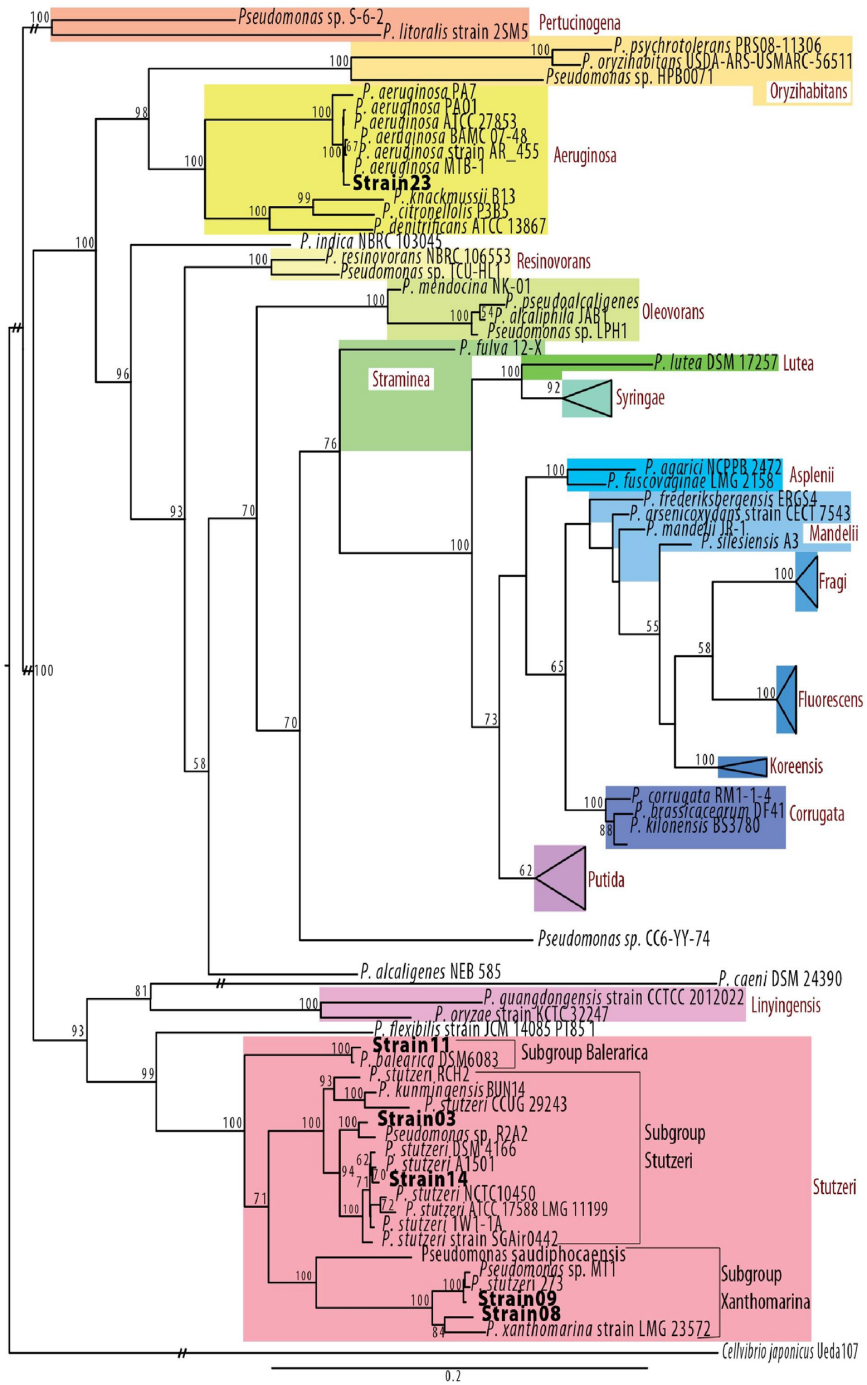
Phylogenetic analysis of Pseudomonas strains. Maximum Likelihood topology of Pseudomonas genus based on the concatenation of 10 conserved proteins with 1000 bootstraps. A total of six strains, representing each of the six subgroups, were used in the tree construction i.e. strains 23, 11, 03, 14, 09, 08. Support values greater than 50% are indicated on the tree. Bar represents 0.2 amino acid substitutions per site. Sequences determined here are indicated in bold. *Cellvibrio japonicus* Ueda107 was used as outgroup. Each clade is colorated based on the group in which it belongs, while the name of the group is indicated in red. Branches with the “//” symbol have been truncated for presentation reasons.

All strains were closely related to six culture representatives and clustered into six subgroups (i.e. Stutzeri 1 and Stutzeri 2, Balearica, Aeruginosa, Xanthomarina 1 and 2). Strains in bold, representing each of the six subgroups, were used in phylogenetic tree construction presented in Fig. 4.

### Phylogeny

Phylogenetic tree of the 21 strains was in agreement with previously published topologies for the genus of *Pseudomonas*^32,33^. The tree was binary without any multifurcations, with the 21 strains falling into two well-known *Pseudomonas* groups, i.e. the *Aeruginosa* and the *Stutzeri* groups (Bootstrap values = 100; Table 1; Fig. 4). From the total 21 strains analyzed here, the *Aeruginosa* group contained the eight deep seawater layer strains (i.e. Strain05, Strain06, Strain07, Strain16, Strain19, Strain20, Strain21 and Strain23) showing similarity of > 98.97% to *P. aeruginosa* MTB-1 and the *Stutzeri* group the remaining ones. Based on Average Nucleotide Identity (ANI) calculations (Table 1), the *Stutzeri* group was further divided into five distinct subgroups (Bootstrap values = 100; Table 1; Fig. 4). The first one included three strains i.e. Strain04, Strain11 and Strain22 from both the surface and deep seawater layer showing high similarity to *P. balearica* DSM_6803 (> 98.51%). The second group consisted of five surface seawater layer strains with > 97.1% sequence similarity to *Pseudomonas sp.* R2A2 (i.e. Strain01, Strain02, Strain03, Strain12, Strain18). The third group included one surface (Strain14) and one deep seawater layer strain (Strain24) with > 97.3% sequence similarity to *P. stutzeri* 1W1-1A. The fourth group is composed of two surface layer strains with > 97.1% similarity to *Pseudomonas sp.* MT-1 (Strain09, Strain10), whereas the last group contained a single strain showing low similarity to its closest culture representative (i.e. Strain08, 88.8% to *P. xanthomarina* LMG 23572; Table 1).

### Pangenome analysis

**Figure 5:**
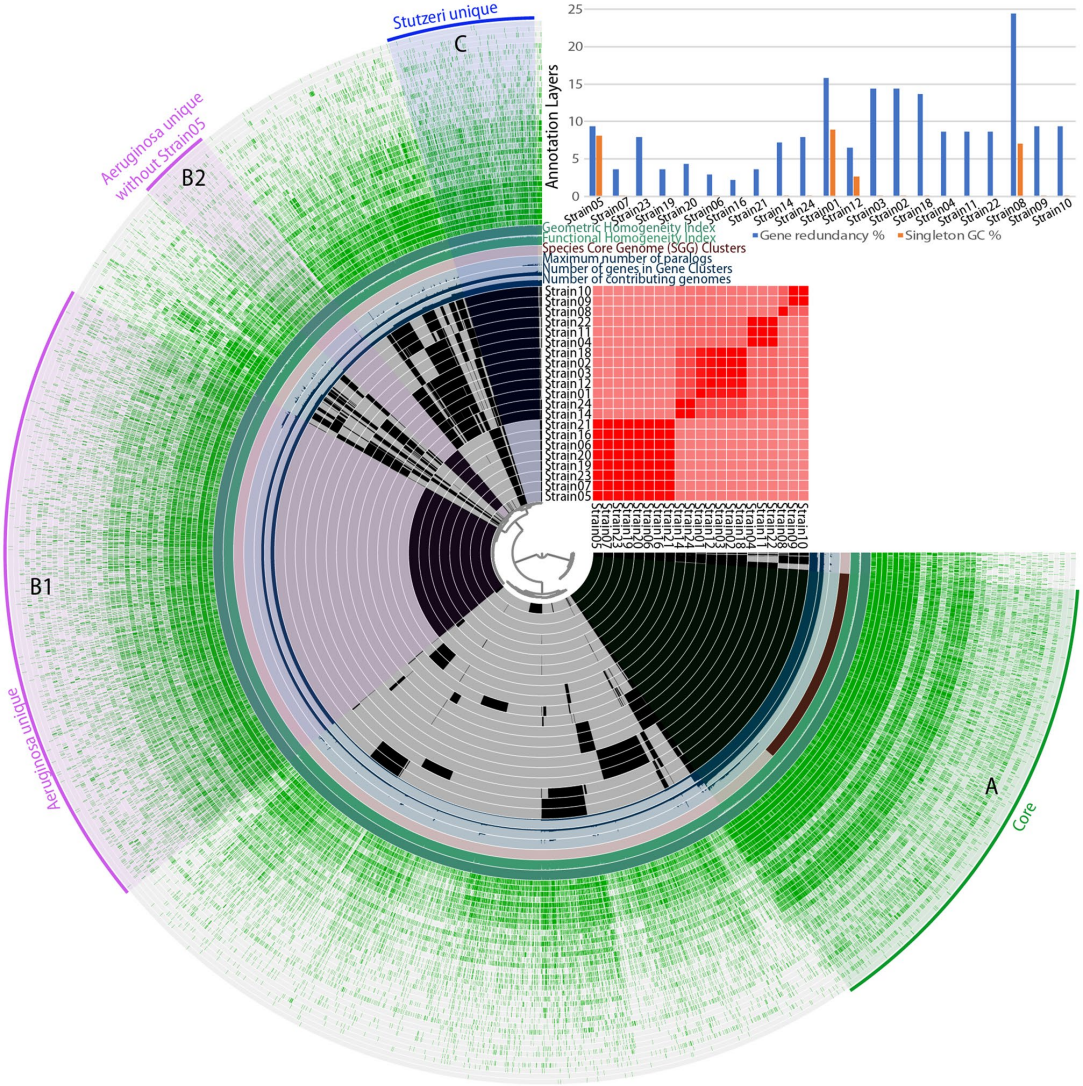
Anvi’o representation of the pangenome of the 21 Pseudomonas genomes. The first 21 layers represent each genome, and the black coloration signifies the existence of a Gene Cluster (GC). To the right is given an ANI percentage identity heatmap of the same genomes. The subsequent 6 layers correspond to various statistics related to the analysis i.e. the number of contributing genomes per GC, number of genes per GC, maximum number of paralogs per GC, Species Core Genome Clusters, Functional Homogeneity Index and Geometric Homogeneity Index. The last 38 layers represent the various annotation sources for each GC and the green coloration signifies the existence of annotation. “A” represents the common GCs to all 21 strains (Core genome). “B1” represents the GCs unique to the *Aeruginosa* group strains and “C” represents the GCs which are unique to the *Stutzeri* group strains. “B2” represents the GCs specific to the *Aeruginosa* group strains but without Strain05 which has no GCs in this bin. The bar chart at the top right represents the gene redundancy (blue bar color) and the singleton GC (orange bar color) per genome.

Gene clusters (GCs) were formed during the pangenome analysis and consisted of CDS from different genomes that were grouped and aligned together. Pangenome analysis identified 10,908 GCs (Supplementary Table S3). A total of 2059 GCs were common to all 21 strains and called “Core” (A in Fig. 5), 2775 GCs were found exclusively to the *Aeruginosa* group strains and called them unique (“Aeruginosa-unique”; B1 in Fig. 5), whereas 643 GCs were found exclusively to all the *Stutzeri* group strains (“Stutzeri-unique”; C in Fig. 5). An additional 325 GCs were unique to 7 out of the 8 strains of the *Aeruginosa* group (i.e. not found in Strain05; B2 in Fig. 5). Functional enrichment analysis resulted in 146 KEGG functions for Core GCs, 11 unique functions for *Aeruginosa* group strains and only 4 unique functions for *Stutzeri* group strains^34–36^. Most KEGG functions for Core GCs were associated with transporters (i.e. 10.4%), as well as DNA repair and recombination proteins (i.e. 4.5%; Supplementary Table S4). The unique GCs for *Aeruginosa* group were related to functions of biotechnological importance, such as geraniol degradation^37^, central carbon metabolism in cancer^38^, betalain biosynthesis^39^, ferroptosis^40^, bisphenol degradation^41^, phenazine biosynthesis^42^ and sphingolipid metabolism^43^. According to Blast2GO functional associations^44^, the vast majority of the core functions were related to an integral component of membrane (i.e. 5.4%), while according to Phobius associations^45^, the majority of the core protein regions were predicted to be extracellular (26.2%), followed by those predicted to be transmembrane (i.e. 17.5%) and then by the cytoplasmic ones (i.e. 16.7%) (data not shown). Remarkable differences between *Aeruginosa* and *Stutzeri* group strains were noticed for GCs, which are related to phages and sigma (σ) factors. More specifically, in *Aeruginosa* group strains, the phage-related GCs were 34 including a hypothetical protein of bacteriophage Pf1, phage-related tail proteins, bacteriophage tail proteins, helix destabilizing proteins of bacteriophage Pf1, P2-like prophage tail proteins, phage tail sheath protein, phage tail assembly chaperone proteins etc. whereas for the *Stutzeri* group were only 4 i.e. the ORF phage PA0727, a phage/conjugal plasmid C-4 type zinc finger protein, a putative bacteriophage protein and a phage holing family protein (Supplementary Table S5). Similarly, the sigma (σ) factors which are involved in the regulation of gene expression were 26 for the *Aeruginosa* group strains and only 7 for the *Stutzeri* ones. The *Aeruginosa* group GCs included the RNA polymerase sigma-70 factor and the sigma-24 subunit of the ECF subfamily, the sigma factor PvdS, the sigma-70 factors family signature 2, the extracytoplasmic function (ECF) sigma factor and the sigma-70 factor Fpvl (Supplementary Table S6). In addition, very few GCs related to transposable elements were found, and these included 4 for *Aeruginosa* group strains, which are linked to transposition, DNA-mediated functions, transposase and inactivated derivates of IS5 family and other transposase and only 2 GCs of transposition-related function for the *Stutzeri* group strains (Supplementary Table S7).

### GCs of antibiotic and multidrug resistance

Mandalakis et al.^4^ reported that the bacterial isolates from Kolumbo crater floor exhibited higher tolerance to antibiotics compared to isolates from surface seawaters. To further ascertain whether there are genetic features that shape this difference between deep and surface seawater microbes we attempted to mine genes from the 21 genomes related to antimicrobial or/and multidrug resistance, i.e. resistance to two or more classes of antibiotics^46^. Following the results of Mandalakis et al.^4^, the strains were grouped based on their phenotypic tolerance to antibiotics, i.e. -into “High Tolerance” and “Low Tolerance” (Table 1) and a functional enrichment comparison of the two groups was performed (Supplementary Table S8). The “High Tolerance” group included all members of the *Aeruginosa* group plus the *Stutzeri* group member Strain08, while the “Low Tolerance” group included the rest 12 strains (Table 1). In this analysis, we focused only on the GC-associated functions that were unique to each group. The “High Tolerance” group comprised 37 unique GCs associated with antimicrobial and multidrug resistance, such as antimicrobial resistance genes, multidrug efflux systems and pumps and transmembrane transporters^47^. In addition, a GC associated with a TetR/AcrR family transcriptional regulator that regulates antibiotic resistance was also identified for the “High Tolerance” group^48^. On the contrary, only 3 GCs associated with antimicrobial and multidrug resistance were found for the “Low Tolerance” group (Supplementary Table S8).

## Discussion

One of the biggest current threats to human health is the rise of antibiotic and multidrug resistant bacteria^1,2^. Microorganisms produce many antimicrobials in nature, they become resistant to the antibiotics they produce, and then, the genes of resistance can be transferred to other non-resistant bacteria^49^. The presence and accumulation of antibiotics in the environment may provide an additional selective pressure for the transmission of AR genes to non-resistant bacteria^2,50^. The possible mechanisms of anthropogenic origin that can lead to the occurrence of AR in marine habitats include (a) coastal runoff of AR bacteria from terrestrial sources and (b) selection for AR due to anthropogenic antibiotic runoff, which challenges native microbes to become resistant^11^.

Our understanding of the environmental factors that may control the AR selection in extreme environments is still limited. In our previous work^4^ we noticed an enhanced co-tolerance to acidity and antibiotics of *Pseudomonas* strains isolated from the deep seawater (430 and 495 m) layers of the hydrothermally active submarine Kolumbo volcano compared to those isolated from the surface seawater (5–90 m). In the present study, we performed whole genome sequencing for 21 of these strains to gain insight into the genetic basis of the enhanced co-tolerance to antibiotics and acidity. A series of tools were used for complete genome assembly and annotation, whereas a total of 28 different databases were used in order to comprehensively assign functions to the predicted genes. The example case presented in Table 2 demonstrates the benefits gained from using multiple annotation sources. This advantage becomes more obvious when annotation for a gene is missing in some databases (grey gaps in Fig. 5). In this example case given in, the annotated CDS most probably represents a 6-pyruvoyl-tetrahydropterin synthase (PTPS), since at least 10 of the annotations point to the same enzyme. PTPS is a well-studied enzyme, and in *P. aeruginosa*, is involved in the biosynthesis of the modified tRNA nucleosides queuosine and archaeosine^51^. This is also supported by the position of the CDS in the genome, i.e. next to the rRNA operon. The annotations from KEGG Function^34–36^ and TIGRFAM agree on this function, while the more general annotations from COG Category and KEGG Pathways are not very informative. By contrast, Reactome and Superfamily seem to give erroneous annotations (biosynthesis of tetrahydrobiopterin). Reactome annotations, in particular, are based on datasets of *Homo sapiens* and are thus expected to not be pertinent for bacteria.

**Table 2:** Functional annotation of a CDS (yellow border in Fig. 3Β).

Annotation source	Accession	Function
Blast2Go	P: GO:0008616; F:GO:0046872; F:GO:0070497	P: queuosine biosynthetic process; F:metal ion binding; F:6-carboxy-5,6,7,8-tetrahydropterin synthase activity
COG Category	H	Coenzyme transport and metabolism
COG Function	COG0720	6-Pyruvoyl-tetrahydropterin synthase
EggNOG	PST_2317	6-Carboxy-5,6,7,8-tetrahydropterin synthase
EggNOGKegg	K01737	queD, ptpS, PTS; 6-pyruvoyltetrahydropterin/6-carboxytetrahydropterin synthase [EC:4.2.3.12 4.1.2.50]
Gene3D-4.2.0	G3DSA:3.30.479.10	6-Pyruvoyl tetrahydropterin synthase/QueD
Interpro-72.0	IPR007115	6-Pyruvoyl tetrahydropterin synthase/QueD family
KEGG Brite; KEGG Pathway	K09180; K09182	–; Protein families: genetic information processing
KEGG Function	K03016	Transfer RNA biogenesis [BR: ko03016]
KEGG Genes	K01737	queD, ptpS, PTS; 6-pyruvoyltetrahydropterin/6-carboxytetrahydropterin synthase [EC:4.2.3.12 4.1.2.50]
PANTHER Protein	PTHR12589:SF7	None
Pfam InterPro	PF01242	6-Pyruvoyl tetrahydropterin synthase
PIRSF	PIRSF006113	6-Pyruvoyl tetrahydropterin synthase [Validated]
Prokka	Ref Seq: ABP79976.1	6-Pyruvoyl tetrahydrobiopterin synthase, putative
Rast FigFam	fig|316.409.peg.704	6-Carboxytetrahydropterin synthase (EC 4.1.2.50); Queuosine biosynthesis QueD, PTPS-I; KEGG_ENZYME:4.1.2.50
Reactome	R-HSA-1474151;	Tetrahydrobiopterin (BH4) synthesis, recycling, salvage and regulation, Homo sapiens
SUPERFAMILY	SSF55620	Tetrahydrobiopterin biosynthesis enzymes-like
TIGRFAM	TIGR03367	queuosine_QueD: queuosine biosynthesis protein QueD

Phylogenetic analysis revealed two distinct and well-known *Pseudomonas* groups, the *Aeruginosa* and the *Stutzeri*. Pangenome analysis revealed that the numbers of phage-related GCs and sigma factors were much higher in *Aeruginosa* group compared to the *Stutzeri* one, whereas only few transposable elements were found in both groups. GCs related to both phages and sigma factors are of exceptional importance, as they provide the mechanisms of adaptation to challenging conditions such as extreme pH values or increased antibiotic concentrations. According to Colomer-Lluch et al.^2^, phages can carry antibiotic resistance genes able to confer resistance to bacterial strains and they may influence the generation of resistance in the environment. In addition, the sigma factors can provide the mechanisms for regulating the expression of large numbers of genes in response to changing environmental conditions, such as a temperature rise or a decline in pH (e.g.^52^). The sigma factors found in *Aeruginosa* group of the present study included almost all sigma factors that have been identified so far in *P. aeruginosa* strains^52^. Their presence in sufficient numbers is a strong indication of the high adaptability of *Aeruginosa* group members to the extreme conditions of the active hydrothermal field of Kolumbo volcano. In addition to sigma factors, the high numbers of GCs related to phages in *Aeruginosa* group provided also strong evidence for the presence of genes associated with antibiotic resistance in the CO_2_-rich acidic water column above the active hydrothermal vents of the Kolumbo volcano.

We further examined all unique GCs in *Pseudomonas* strains of high co-tolerance to antibiotics and acidity. Interestingly, all of them were associated with antimicrobial and multidrug resistance. The identified GCs included antimicrobial resistance genes, multidrug efflux systems and pumps and transmembrane transporters. Interestingly, that was not the case for the low tolerant *Pseudomonas* strains implying that strains from the acidic seawater layers above the active area of Kolumbo volcano, carry antimicrobial resistance mechanisms. Bacterial multidrug efflux pumps are antibiotic resistance determinants present in all microorganisms. According to Blanco et al.^47^ multidrug efflux pumps are ancient elements encoded in bacterial genomes long before the recent use of antibiotics for human and animal therapy. These elements can extrude, beside antibiotics, a wide range of non-antibiotic substrates, such as heavy metals (e.g. Cu, Hg, As), organic pollutants and others. The submarine Kolumbo volcano is characterized by a unique metal enrichment of hydrothermal spires and mounds^26^. Such metal enrichment (e.g. Sb, Tl, Ag, As, Hg, Pb, Cu) may have provided selective pressure for the maintenance of resistance mechanisms in the acidic water above the active hydrothermal vents of the Kolumbo crater. These mechanisms are mostly processed by membrane proteins which limits the intracellular access to antibiotics^53^ and allows the increased tolerance of *Pseudomonas* strains from Kolumbo to a series of stressors such as acidity and antibiotics. The present study provided strong evidence that the CO_2_-rich acidic seawater of the Kolumbo volcano serve as a pool of microorganisms carrying GCs for multidrug efflux-mediated systems and pumps and is an ideal natural laboratory to understand the physicochemical factors selective pressure that shape the antibiotic resistome. Additional research is needed to further our insights into the extent to which extreme ecosystems are reservoirs of resistance mechanisms across the globe.

## Materials and methods

### Strains characteristics

A total of 21 bacterial strains previously isolated from surface and deep seawater samples of submarine Kolumbo volcano were used in the present study^4^. Nine of the strains were isolated from surface seawater (i.e. 1 from 5 m, 4 from 20 m, 1 from 45 m, 3 from 90 m) and the rest 12 strains were isolated from deeper seawater (i.e. 1 from 430 m and 11 from 495 m). Mandalakis et al.^4^ performed quantitative testing of all bacterial strains susceptibility to various stressors including pH, heavy metals [i.e. As(III): arsenic; Sb(III): antimony; Sr(II): strontium and Hg(II): mercury] and six commonly used antibiotics (Amp: ampicillin; Eryth: erythromycin; Cipr: ciprofloxacin; Cef: cefuroxime; Tetr: tetracycline; Chlr: chloramphenicol). Based on their phenotypic traits to these stressors, strains were separated into two distinct groups, i.e. the high tolerance and the low tolerance groups to acidity, antibiotics and heavy metals (Table 1; Supplementary Table S1). Cell stocks of all bacterial strains are stored in 50% glycerol at − 80 °C at IMBBC-HCMR microbial strain collection and they were used for cultivation, genomic DNA isolation and whole-genome sequencing.

### Cultivation, DNA extraction, library construction and sequencing

Fresh bacterial cultures were prepared from cell stocks in 5 mL of marine broth medium (Conda, Pronadisa, Madrid, Spain), incubated at 37 °C overnight and used for DNA extraction. Extraction of genomic DNA was performed using the Wizard Genomic DNA Purification kit (Promega, WI, USA) following the instructions provided by the manufacturer. Purified DNA was quantified in a microplate reader (TECAN Infinite F200 Pro, Tecan Trading AG, Männedorf, Switzerland) using Quanti-iT PicoGreen dsDNA assay kit (Fisher Scientific, New Hampshire, USA). Two micrograms (µg) of high molecular weight DNA from each sample was acoustically sheared in a Covaris S220 sonicator (Covaris, Inc., Massachusetts, USA), with a target size of 550 bp. Illumina TruSeq PCR-free libraries were constructed according to the manufacturer’s instructions. The libraries were sequenced in-house on an Illumina MiSeq using 2*300 bp paired-end sequencing.

### Genome assembly and annotation

A series of tools were used for complete genome assembly and annotation. Details are provided in Supplementary file (Supplementary Table S9). The different steps included quality filtering of sequences (using FastQC, Fastp v.0.19.5 and BBtools suite v.38.08), assembly and contamination treatment (using KmerGenie v.1.7016 and Spades v.3.13.0), filtering of scaffolds and contamination (Barrnap v0.9, Blastn v2.8.1+, Contig-Layout-Autheticator pipeline, Kraken 2 v.2.0.7-beta), assembly correction (using Abyss-sealer v.2.1.0, Pilon v.1.23 and Blastn v.2.8.1+), assessment (using CheckM v.1.0.12, Quast v.5.0.2, Busco v.3.1.0, OrthoDB v9, anvi-run-hmms and Anvi’o v.5.4) and annotation (using prokka_database_maker to create a *Pseudomonas* genus database for Prokka, Prokka v.1.13.3 pipeline as input for annotation, GeneMarkS-2 to predict genes, Interproscan v.5.33 for functional annotation against a number of databases; Supplementary Materials and Methods; Supplementary Table S9).

### Phylogenetic analysis

We selected a series of reference strains for the phylogenetic analysis by using the highest ANI similarity results with the sequenced strains of the present study. All reference strains were retrieved from the Genome Taxonomy Database Release 03-RS86^54^. Fasta files of the selected genomes were downloaded either from Pseudomonas Genome Database or NCBI RefSeq. All downloaded fasta files where either complete genomes or had at most 10 contigs. *Cellvibrio japonicus*, a closely related strain to *Pseudomonas* genus, was used as an outgroup^55^. We used the program anvi-script-reformat-fasta to reformat fasta headers, and the output was used with program anvi-gen-contigs-database to create an Anvi’o contig database for each of the 93 strains selected for our analysis. Program anvi-run-hmms was used to store HMM hits of Busco orthologs from OrthoDB v9 Gamma-Proteobacteria database in the anvi'o contigs database. We used the program anvi-get-sequences-for-hmm-hits to select single copy genes which existed in all genomes and aligned the amino acid sequences separately with muscle v.3.8.31. For each of the amino-acid fasta multiple sequence alignments (MSAs) we removed all gaps and renamed fasta headers to make them shorter with an in-house script. Prank v.170427^56^ was used to align each fasta file with the +F option. We inspected each MSA manually with Mega v.10.0.5^57^ and selected 10 amino-acid single copy gene MSAs based on phylogenetic information for downstream analyses. Guidance2^58^ was used with 200 bootstrap repeats to detect unreliable MSA regions and unreliable columns with confidence score below 0.93 were removed. Mega v.10.0.5 was used to inspect and manually trim each MSA if needed.

MEGA-CC v.10.0.5 was used to create a maximum likelihood (ML) tree for each MSA. The program fasta2relaxedPhylip.pl from the Phylogenomic suite^59^ was used to turn each fasta MSA into phylip format. We ran CodeML from the PAML v.4.9 suite^60^ with input each phylip MSA and starting tree the corresponding ML tree, to get an amino acid rate matrix for each gene. In addition, ProtTest v.3.4.2^61^ was used to select the best-fit models for each amino acid MSA. PhyML v.3.3.20190321^62^ was used to construct an ML starting tree from the fasta MSA files. We used the FreeRate model of PhyML for the variation across sites with 4 rate categories for each MSA, the custom amino acid substitution model found by CodeML, estimation of amino acid frequencies from data and different branch length estimation for each MSA. ElConcatenero3^63^ was used to convert and concatenate the fasta MSA files into phylip format. RAxML-NG v.0.9.0^64^ was used to construct an ML tree with starting tree the one constructed by PhyML, model selected for each partition separately, based on the Prottest results, 1000 bootstraps and input data the phylip MSA from ElConcatenero.

### Pangenome analysis

For the pangenome analysis we followed the Anvi’o workflow as it is described by Delmont and Eren (2018)^65^. Anvi’o is an open-source, community-driven analysis and visualization platform for microbial-omics which is available from http://merenlab.org/software/anvio/. We generated an Anvio’s profile database by profiling *Pseudomonas* genomes during which Prodigal v.2.6^31^ identified open reading frames, importing annotations from other databases, annotating genes with functions by searching them against the Clusters of Orthologous Groups (COGs) using blastp v.2.8.1+, removing weak hits with the minbit heuristic^66^, clustering with MCL v.14–137^67^ and aligning gene clusters with muscle v.3.8.31^68^. For the Average Nucleotide Identity (ANIm) calculations we used the programs anvi-compute-ani and NUCmer included in the suite MUMmer v.4.0.0beta2^69^. The program anvi-get-enriched-functions-per-pan-group from Anvi’o v.5.5 was used in order to perform functional enrichment analysis of the *Aeruginosa* and the *Stutzeri* groups. The enrichment analysis allows to statistically quantify how much a functional annotation is unique to the genomes that belong to a specific group versus all other genomes in the pangenome. We defined “core” genes clusters that were systematically detected in all analysed genomes while gene clusters that systematically were detected only in a group or strain were defined as “unique” to that group or strain.

The full reproducible code for all the analyses presented here is available in https://github.com/pbravakos/Pseudomonas_Kolumbo.

## Supplementary Information

Supplementary Information
