# Supplementary material for: Genomic adaptation of Pseudomonas strains to acidity and antibiotics in hydrothermal vents at Kolumbo submarine volcano, Greece
Source: Sci Rep. 2021 Jan 14;11:1336. doi: 10.1038/s41598-020-79359-y (PMC7809023; doi:10.1038/s41598-020-79359-y)
Supplement: Supplementary file 1 — Supplementary Information [file 41598_2020_79359_MOESM1_ESM.docx]

**SUPPLEMENTARY DATA**

# Genomic adaptation of *Pseudomonas* strains to acidity and antibiotics in hydrothermal vents at Kolumbo submarine volcano, Greece

Panos Bravakos^1^, Manolis Mandalakis^1^, Paraskevi Nomikou^2^, Thekla I. Anastasiou^1^, Jon Bent Kristoffersen^1^, Melanthia Stavroulaki^1^, Stephanos Kilias^2^, Georgios Kotoulas^1^, Antonios Magoulas^1^, Paraskevi N. Polymenakou^*,1^

^1^Institute of Marine Biology, Biotechnology and Aquaculture, Hellenic Centre for Marine Research (IMBBC-HCMR), Gournes Pediados, Heraklion Crete, Greece

^2^Department of Geology and Geoenvironment, National Kapodistrian University of Athens, Greece

***Corresponding author:** E-mail: [polymen@hcmr.gr](mailto:polymen@hcmr.gr)

## Materials and Methods

## Genome Assembly and Annotation

## Quality Filtering

Sequences were inspected with FastQC v.0.11.8^1^ to check their quality. BBsplit from the BBtools suite v.38.08[^2^](https://www.zotero.org/google-docs/?BocRMF) was used to filter out remnant PhiX sequences. Fastp v.0.19.5[^3^](https://www.zotero.org/google-docs/?3RfNnW) was used for quality filtering. Fastp cannot produce singleton reads, meaning, it filters out either both paired reads or none of them. In order to be able to trim each read pair separately, we used PRINSEQ-lite v.0.20.4[^4^](https://www.zotero.org/google-docs/?syPaXj). BBnorm from the BBtools suite v.38.08 was used to filter out low-depth kmers and finally BBmerge from the same suite was used to merge paired end reads.

## Assembly

KmerGenie v.1.7016^5^ was used to estimate the best k-mer lengths for genome de novo assembly. We run KmerGenie multiple times, each time with a different largest k-mer size to consider, potentially having each time a different output. Spades v.3.13.0[^6^](https://www.zotero.org/google-docs/?jlFMeq) was used to assemble de novo the short reads to scaffolds with the kmer sizes proposed by KmerGenie.

##

## Contamination treatment

For strains that had contaminated scaffolds, further steps were included in the pipeline to address the problem. In Bandage[^7^](https://www.zotero.org/google-docs/?QGPoEy) we inspected the Graphical Fragment Assembly (GFA) output of Spades and we selected sequences of interest based on orientation and best BLAST hits[^8^](https://www.zotero.org/google-docs/?Km2Zgw). We downloaded selected sequences to a fasta file for each assembly. We used BWA-MEM v.0.7.17-r1188[^9^](https://www.zotero.org/google-docs/?faZtPz) to map the original fastq reads to the fasta file downloaded from Bandage and retrieved only the fastq reads that composed the genome of interest. BBmerge was used to merge paired end fastq reads filtered by BWA. KmerGenie was used to search the selected fastq reads for the best kmer and finally Spades was used to assemble the filtered fastq reads using as kmers the kmers selected by KmerGenie.

##

## Scaffolding and Contamination filtering

Barrnap v0.9[^10^](https://www.zotero.org/google-docs/?N204XD) was used to predict the location of ribosomal RNA genes in genomes. Blastn v.2.8.1+ was used to search barrnap output against the Reference Sequence (RefSeq) nucleotide database at NCBI[^11^](https://www.zotero.org/google-docs/?FHyBaH). The best BLAST results were downloaded and kept as reference strains for scaffolding. Contig-Layout-Authenticator (CLA)[^12^](https://www.zotero.org/google-docs/?GzB0ki) pipeline was used to scaffold Spades output. Kraken 2 v.2.0.7-beta^13^ was used against standard Kraken 2 database to classify CLA output scaffolds. Only scaffolds classified by Kraken in the *Pseudomonadaceae* family were kept for downstream analyses. No unclassified scaffolds were observed.

##

## Assembly Correction

Abyss-sealer v.2.1.0[^14^](https://www.zotero.org/google-docs/?4gPy8N) was used to close gaps within scaffolds, with a bloom filter created by the fastp output fastq reads. Pilon v.1.23[^15^](https://www.zotero.org/google-docs/?AYMt4z) was used to improve the assembly with bam files created by the Prinseq output fastq reads. Blastn v.2.8.1+ was used to search Pilon scaffolds against the GenBank nucleic acid sequence database [^16^](https://www.zotero.org/google-docs/?vLKnxL). Scaffolds, whose BLAST hits were not related to Pseudomonas, were removed. The best BLAST hits were downloaded and kept as Reference strains for all downstream analyses.

##

## Assembly Assessment

CheckM v.1.0.12 [^17^](https://www.zotero.org/google-docs/?8s560g), Quast v.5.0.2 [^18^](https://www.zotero.org/google-docs/?9x20aZ) and Busco v.3.1.0 search against the 452 single copy orthologs from the OrthoDB v9 [^19^](https://www.zotero.org/google-docs/?7Gacui) Gamma-Proteobacteria database were used to assess the assembly. Kaiju^20^ gene-level taxonomy searched against NCBI’s RefSeq. The program anvi-run-hmms was used to store hmm hits for single copy genes for Archaea[^21^](https://www.zotero.org/google-docs/?j4kIGI), Bacteria[^22^](https://www.zotero.org/google-docs/?Tz15TW), Protista into Anvi’o v.5.4[^23^](https://www.zotero.org/google-docs/?Im9n2K) to assess the contamination levels.

##

## Assembly Annotation

We used prokka_database_maker^24^ to create a *Pseudomonas* genus database for Prokka^25^ from NCBI. Subsequently, we run the Prokka v.1.13.3 pipeline specifying this database as input for annotation. Prokka predicted protein-coding features with Prodigal v.2.6[^26^](https://www.zotero.org/google-docs/?1kGhfL), tRNA genes with ARAGORN v.1.2[^27^](https://www.zotero.org/google-docs/?MyaDW7), ribosomal RNA genes with Barrnap v.0.9, and non-coding RNAs with Infernal v.1.1.2[^28^](https://www.zotero.org/google-docs/?2x4223). Besides Prodigal, we also used GeneMarkS-2[^29^](https://www.zotero.org/google-docs/?gfaOrd) to predict coding sequences (CDS) (Supplementary Fig. 1). Most of the CDS predicted by GeneMarkS2 were almost the same to the CDS predicted by Prodigal, but with a minor change in the starting position. This difference could probably be attributed to the different gene calling algorithms used. In a few cases, there was a novel CDS introduced by GeneMarkS2. Sometimes, the introduced CDS was found in places with reduced coverage that made the new CDS. For this reason, we decided to rely our analysis almost exclusively on the CDS found by Prodigal. All gene calls found by Prodigal were parsed into Anvi’o but especially for GenemarkS2 gene calls, parsing happened only when the gene calls were different from the ones Prodigal predicted. We run Interproscan v.5.33[^30^](https://www.zotero.org/google-docs/?RQ6n3y) for functional annotation against the following databases (Supplementary Table 2): Interpro-72.0[^31^](https://www.zotero.org/google-docs/?K4q7TM), CDD-3.16[^32^](https://www.zotero.org/google-docs/?077Qn3), Gene3D-4.2.0[^33^](https://www.zotero.org/google-docs/?5un6XW), Hamap-2018_03[^34^](https://www.zotero.org/google-docs/?u2Qpjo), MobiDBLite-2.0[^35^](https://www.zotero.org/google-docs/?jxEL3A), PANTHER-12.0[^36,37^](https://www.zotero.org/google-docs/?AZsEID), Pfam-32.0[^38^](https://www.zotero.org/google-docs/?VNEjr2), Phobius-1.01[^39^](https://www.zotero.org/google-docs/?spJ5K2), PIRSF-3.02[^40^](https://www.zotero.org/google-docs/?SwIPfp), PRINTS-42.0 [^41^](https://www.zotero.org/google-docs/?cIyjC9), ProDom-2006.1[^42^](https://www.zotero.org/google-docs/?VSGd87), ProSitePatterns-2018_02[^43^](https://www.zotero.org/google-docs/?gtbIky), SFLD-4[^44^](https://www.zotero.org/google-docs/?BPd6kT), SMART-7.1[^45^](https://www.zotero.org/google-docs/?5RcaiQ), SUPERFAMILY-1.75[^46^](https://www.zotero.org/google-docs/?fFsYO7), TIGRFAM-15.0[^47^](https://www.zotero.org/google-docs/?srHJFM), Reactome[^48^](https://www.zotero.org/google-docs/?tnrsqu), MetaCyc[^49^](https://www.zotero.org/google-docs/?M5yBk8), KEGG[^50^](https://www.zotero.org/google-docs/?9VltH8)^-52^. For the PANTHER IDs predicted by Interproscan we run the PANTHER Generic Mapping workflow[^36^](https://www.zotero.org/google-docs/?bUcA82) having as reference proteome the genus *Pseudomonas aeruginosa*, and extracted the following annotation classes: PANTHER Pathway and PANTHER GO-Slim. For the PIRSF IDs predicted by Interproscan we run the Batch Retrieval service of PIRSF database and extracted the annotations. For the SMART IDs predicted by Interproscan we downloaded the SMART domain descriptions and extracted the annotations. For the SUPERFAMILY IDs found by Interposcan we downloaded the functional annotation of Structural Classification of Proteins database v.1.73 and extracted the annotations. For the Gen3D IDs predicted by Interproscan we downloaded the name description of each node in the CATH hierarchy and extracted the annotations. For the Reactome IDs predicted by Interporscan we downloaded the Reactome pathways and extracted the annotations. For the MetaCyc IDs predicted by Interporscan we downloaded all pathways of MetaCyc and extracted the annotations. For KEGG accessions found by Interproscan we downloaded KEGG Pathway Maps and KEGG Enzyme hierarchy files and extracted the annotations. SignalP v4.1[^53^](https://www.zotero.org/google-docs/?YCZqtK) was used for prediction of signal peptides. TMHMM v.2.0c[^54^](https://www.zotero.org/google-docs/?MmBS2i) was used for transmembrane helices prediction. LipoP 1.0a[^55^](https://www.zotero.org/google-docs/?AkTnjl) was used to predict lipoproteins. BlastKoala [^56^](https://www.zotero.org/google-docs/?EvBshS), was used to search for each gene of the genomes against the KEGG database. Antismash v.4.2.0 [^57^](https://www.zotero.org/google-docs/?MgTOfj) was used to search for secondary metabolites and the annotations from secondary metabolite clusters of orthologous groups (smCOGs) were extracted from the Antismash output. EggNOGmapper v.1.0.3[^58^](https://www.zotero.org/google-docs/?40T7KK) was used to annotate the genomes against the EggNOG v.5.0 database with DIAMOND[^59^](https://www.zotero.org/google-docs/?wqV4W6). Genomes were also annotated in the Rast Server[^60^](https://www.zotero.org/google-docs/?7DzZsN) using the gene calls predicted by Prodigal against the SEED database[^61^](https://www.zotero.org/google-docs/?Xiugm8). Blastp was used to search all gene calls predicted by Prodigal against the GenBank nucleic acid sequence database. Interproscan and blastp outputs were parsed into Blast2Go[^62^](https://www.zotero.org/google-docs/?LsrwzB) in order to annotate the genomes with Gene Ontology terms. Anvi’o was used to search against the COG database[^63^](https://www.zotero.org/google-docs/?hw5G2m). All annotations were parsed into Anvi’o with an in-house script by using the program anvi-import-functions and stored in an Anvi’o contig database, splitting the contigs longer than 20,000 base pairs into smaller ones. The program anvi-profile was used to create an Anvi’o profile database with input the bam files created by BWA-MEM for paired, singleton and merged reads and to search for single-nucleotide variants (SNVs). Prediction of antibiotic resistance genes was performed using RGI v.5.1.0[^64^](https://www.zotero.org/google-docs/?X24LHA). Finally, when needed to search for structures of specific CDSs in the PDB database we used MODELLER v.9.21[^65^](https://www.zotero.org/google-docs/?axeZD3). Although a large number of tools and databases were used (Supplementary Table 2), significant results were obtained by KEGG, Blast2Go and Phobius. In addition, phages related GCs were retrieved from ProDom, TIGRFAM, Prokka, COG_FUNCTION, Interpro, PfamInterPro, Rast_FigFam, SUPERFAMILY and EGGNOG (Supplementary Tables 2 & 5). Sigma factors were obtained from EggnogKegg, EGGNOG, KEGG_Genes, Prokka, ProSitePatterns and RastFigFam (Supplementary Tables 2 & 6). Transposases were retrieved from Blast2Go and COG_FUNCTION (Supplementary Tables 2 & 7). GCs associated with antimicrobial and multidrug resistance were retrieved from KEGG_Function, EggnogKegg, TIGRFAM, PRINTS, KEGG_Genes, Blast2Go, PANTHER_GO-Slim_Cellular, Interpo_GoTerms, Prokka, COG_FUNCTION, RastFigFam, Gene3D, SUPERFAMILY, EGGNOG and PfamInterPro (Supplementary Tables 2 & 8).

##

## Supplementary Results and Discussion

## Genome Analysis

Single Nucleotide variants (SNVs) are an indication of how reliable the assembly is at each base position. Possible explanations for the existence of SNVs include the presence of multiple short repetitive sequences e.g. in a TIGR02444 family protein transcriptional factor found in *P. aeruginosa* genomes. It is worth noting that *P. aeruginosa* has lower AT content (33.6 % in our strains) compared to other *Pseudomonas* genomes and this may result from the higher level of local repeats[^66^](https://www.zotero.org/google-docs/?7Tl6dW). An additional explanation for the SNVs is the tendency of the assembly process to collapse repetitive regions, e.g., phenazine biosynthesis operons, which were previously reported to be present in two copies in *P. aeruginosa* genomes[^67^](https://www.zotero.org/google-docs/?Y3xTfs), but identified in one copy in our assemblies. Nevertheless, SNVs were also detected even when the assembly process resolved some of the multiple copies operons, such as rRNA operons. For example, in Strain03 where three distinct rRNA operons were identified, SNVs can still be found in the 16S rRNA gene, accompanied by a peak in the genome coverage (Fig. 3 of the main text). Moreover, a partial 16S rRNA was close to one of these rRNA operons indicating reads that were not resolved properly (Fig. 4 of the main text). Finally, some of the SNVs could possibly be explained by sequencing errors, especially in cases when there was not an increase in the coverage of the SNV regions compared to adjacent regions.

Some of the final assembled genomes, had contamination that could not be completely removed. Strain08 for example had probably the most leftover contamination as is evident from the 85 Busco duplicates (Supplementary Table 2) and the level of contamination (23.4%) in Fig. 2 of the main text. The best blast hit for Strain08 is *P. xanthomarina* strain LMG 23572 (LT629970) (Table 1 of the main text) which is about 4.3 Mb while Strain08 is 5.8 Mb (Fig. 2 of the main text). The difference is quite significant and if we take into consideration the high (87%) ANI similarity between the two strains, then the best explanation for the genome size difference is contamination. This contamination seems to come from highly similar strains since the strain heterogeneity is also high (95%) and probably for this reason it was impossible for our pipeline to successfully deal with it. It is also the only strain that had a lower N50 in the final scaffolds (1.05 Mb) compared to the Spades scaffolds (1.07 Mb) although the number of unidentified bases (N’s) were drastically removed in the final output and additionally the N75 metric was significantly better in the final output (1.02 Mb compared to 0.35 Mb in the initial assembly). Strain08 also has the highest gene redundancy (24,5%) among all strains and furthermore 6.6% of the total gene clusters found in its genome were singletons i.e. found only in this genome (Fig. 5 of the main text). The last two metrics (gene redundancy and singleton percentage) are not an indication of contamination per se, but the elevated percentages could possibly be explained by contamination levels. For example the high gene redundancy could be explained by the fact that the contamination source is derived from close relative strain(s), while the high percentage of singleton GCs could be explained by the level of contamination (23.4%). Similar arguments apply for Strain05, Strain01 and Strain13 which have various levels of contamination in the final scaffolds. Strain25 is a special case since it doesn’t seem to have any significant level of contamination but it has the largest singleton percentage (11.5%) (Fig. 5 of the main text), the lowest level of completion (99%) (Fig. 2 of the main text) and the genome size is slightly bigger than its close relatives (Strain18 and Strain13).

Taxonomy at the level of the contig splits, although not very informative for inspecting genome-level contamination, was useful for taxonomic assignments of scaffold segments (splits) that did not appear to be *Pseudomonadaceae* (Fig. 3 of the main text). When these splits were assigned to a more distant clade than the one expected by the taxonomy of the genome they come from, this indicated the existence of phage or transposon related regions. For example, in Fig. 3A of the main text, the green color in the outer layer depicts a split that was taxonomically assigned to *Neorhizobium galegae* and that encodes phage related proteins such as phage capsid and phage tail proteins. The yellow color in Fig. 4B of the main text depicts a split assigned to *Aeromonas* sp. and contains transposases and integrases.

On the other hand, splits that contained putative plasmid genes were often taxonomically assigned to clades phylogenetically close to the *Pseudomonas* genus e.g. in the Proteobacteria class. Analysis with the generic bacterial single copy genes[^22^](https://www.zotero.org/google-docs/?XWP4Gb) in Anvi’o[^68^](https://www.zotero.org/google-docs/?F61T1G) showed that completeness was equal or higher than 85.6% for all strains except Strain08 for which the completeness was 80.6%. Analysis in CheckM [^17^](https://www.zotero.org/google-docs/?hsKoPv) with the phylogeny aware single copy genes showed completeness equal or higher than 98.9% for all genomes. Analysis in Busco[^69^](https://www.zotero.org/google-docs/?PqvtF0) with the phylogeny aware dataset showed completeness equal or higher than 99.7%. Genomes lacked at most one Busco Ortholog Group (OG), the number of duplicate OGs was low, except for Strain08, while only one fragmented OG was identified in Strain13 (Supplementary Table 2).

## Phylogenetic Analysis

Based on the literature two intrageneric groups or lineages have been described for the *Pseudomonas* genus, the one is named “Fluorescens lineage” and the second one “Aeruginosa lineage”[^70^](https://www.zotero.org/google-docs/?avxk3E). Our results confirm the existence of both lineages, with the Aeruginosa group containing eight out of the 21 analyzed strains. All these eight strains were derived from the active area of Kolumbo which all showed high tolerance to acidity, antibiotics and heavy metals in the study by Mandalakis et al. ^71^. These were Strain05, Strain06, Strain07, Strain16, Strain19, Strain20, Strain21 and Strain23 sharing an ANI higher than 98.8%. Their closest relative was *P. aeruginosa* MTB-1.

## References

1. [Andrews, S. FastQC A Quality Control tool for High Throughput Sequence Data.](https://www.zotero.org/google-docs/?QWD4qM) (2014).
2. [Bushnell, B., Rood, J. & Singer, E. BBMerge – Accurate paired shotgun read merging via overlap. *PLOS ONE* **12**, e0185056](https://www.zotero.org/google-docs/?QWD4qM) (2017).
3. [Chen, S., Zhou, Y., Chen, Y. & Gu, J. fastp: an ultra-fast all-in-one FASTQ preprocessor. *Bioinformatics* **34**, i884–i890 (2018).](https://www.zotero.org/google-docs/?QWD4qM)
4. [Schmieder, R. & Edwards, R. Quality control and preprocessing of metagenomic datasets. *Bioinforma. Oxf. Engl.* **27**, 863–864 (2011).](https://www.zotero.org/google-docs/?QWD4qM)
5. [Chikhi, R. & Medvedev, P. Informed and automated k-mer size selection for genome assembly. *Bioinformatics* **30**, 31–37 (2013).](https://www.zotero.org/google-docs/?QWD4qM)
6. [Bankevich, A., *et al.* 2012. SPAdes: a new genome assembly algorithm and its applications to single-cell sequencing. *J. Comput. Biol. J. Comput. Mol. Cell Biol.* **19**, 455–477 (2012).](https://www.zotero.org/google-docs/?QWD4qM)
7. [Wick, R. R., Schultz, M. B., Zobel, J. & Holt, K. E. Bandage: interactive visualization of de novo genome assemblies. *Bioinformatics* **31**, 3350–3352 (2015).](https://www.zotero.org/google-docs/?QWD4qM)
8. [Camacho, C., *et al.* BLAST+: architecture and applications. BMC Bioinformatics 10:421 (2009).](https://www.zotero.org/google-docs/?QWD4qM)
9. [Li, H. Aligning sequence reads, clone sequences and assembly contigs with BWA-MEM. *ArXiv Prepr.* **ArXiv**, 13033997 (2013).](https://www.zotero.org/google-docs/?QWD4qM)
10. Seemann, T. barrnap 0.9 : rapid ribosomal RNA prediction. Available from: https://github.com/tseemann/barrnap (2018).
11. [O’Leary, N. A., *et al.* Reference sequence (RefSeq) database at NCBI: current status, taxonomic expansion, and functional annotation. *Nucleic Acids Res.* **44**, D733–D745 (2015).](https://www.zotero.org/google-docs/?QWD4qM)
12. [Shaik, S., Kumar, N., Lankapalli, A. K., Tiwari, S. K., Baddam, R. & Ahmed, N. Contig-Layout-Authenticator (CLA): A combinatorial approach to ordering and scaffolding of bacterial contigs for comparative genomics and molecular epidemiology. *PLOS ONE* **11**, e0155459 (2016).](https://www.zotero.org/google-docs/?QWD4qM)
13. [Wood, D. E. & Salzberg, S. L. Kraken: ultrafast metagenomic sequence classification using exact alignments. *Genome Biol.* **15**, R46 (2014).](https://www.zotero.org/google-docs/?QWD4qM)
14. [Jackman, S. D., *et al.* ABySS 2.0: resource-efficient assembly of large genomes using a Bloom filter. *Genome Res.* **27**, 768–777 (2017).](https://www.zotero.org/google-docs/?QWD4qM)
15. [Walker, B. J., *et al.* Pilon: An integrated tool for comprehensive microbial variant detection and genome assembly improvement. *PLOS ONE* **9**, e112963 (2014).](https://www.zotero.org/google-docs/?QWD4qM)
16. [Benson, D. A., *et al.* 2012. GenBank. *Nucleic Acids Res.* **41**, D36–D42 (2012).](https://www.zotero.org/google-docs/?QWD4qM)
17. [Parks, D. H., Imelfort, M., Skennerton, C. T., Hugenholtz, P. & Tyson, G. W. CheckM: assessing the quality of microbial genomes recovered from isolates, single cells, and metagenomes. *Genome Res.* **25**, 1043–1055 (2015).](https://www.zotero.org/google-docs/?QWD4qM)
18. [Gurevich, A., Saveliev, V., Vyahhi, N. & Tesler, G. QUAST: quality assessment tool for genome assemblies. *Bioinformatics* **29**, 1072–1075 (2013).](https://www.zotero.org/google-docs/?QWD4qM)
19. [Zdobnov, E. M., *et al.* OrthoDB v9.1: cataloging evolutionary and functional annotations for animal, fungal, plant, archaeal, bacterial and viral orthologs. *Nucleic Acids Res.* **45**, D744–D749 (2017).](https://www.zotero.org/google-docs/?QWD4qM)
20. [Menzel, P., Ng, K. L. & Krogh, A. Fast and sensitive taxonomic classification for metagenomics with Kaiju. *Nat. Commun.* **7**, 11257 (2016).](https://www.zotero.org/google-docs/?QWD4qM)
21. [Rinke, C., *et al.* Insights into the phylogeny and coding potential of microbial dark matter. *Nature* **499**, 431 (2013).](https://www.zotero.org/google-docs/?QWD4qM)
22. [Campbell, J. H., *et al.* UGA is an additional glycine codon in uncultured SR1 bacteria from the human microbiota. *Proc. Natl. Acad. Sci.* **110**, 5540 (2013).](https://www.zotero.org/google-docs/?QWD4qM)
23. [Eren, A. M., *et al.* Anvi’o: an advanced analysis and visualization platform for ‘omics data.van Gulik W, editor. *PeerJ* **3**, e1319 (2015).](https://www.zotero.org/google-docs/?QWD4qM)
24. Ehrlich, R. prokka_database_maker. Available from: <https://github.com/rehrlich/> prokka_database_maker (2016).
25. [Seemann, T. Prokka: rapid prokaryotic genome annotation. *Bioinformatics* **30**, 2068–2069 (2014).](https://www.zotero.org/google-docs/?QWD4qM)
26. [Hyatt, D., Chen, G.-L., LoCascio, P. F., Land, M. L., Larimer, F. W. & Hauser, L. J. Prodigal: prokaryotic gene recognition and translation initiation site identification. *BMC Bioinformatics* **11**, 119 (2010).](https://www.zotero.org/google-docs/?QWD4qM)
27. [Laslett, D. & Canback, B. ARAGORN, a program to detect tRNA genes and tmRNA genes in nucleotide sequences. *Nucleic Acids Res.* **32**, 11–16 (2004).](https://www.zotero.org/google-docs/?QWD4qM)
28. [Nawrocki, E. P. & Eddy, S. R. Infernal 1.1: 100-fold faster RNA homology searches. *Bioinformatics* **29**, 2933–2935 (2013).](https://www.zotero.org/google-docs/?QWD4qM)
29. [Lomsadze, A., Gemayel, K., Tang, S. & Borodovsky, M. Modeling leaderless transcription and atypical genes results in more accurate gene prediction in prokaryotes. *Genome Res.* **28**, 1079–1089 (2018).](https://www.zotero.org/google-docs/?QWD4qM)
30. [Jones. P., *et al.* InterProScan 5: genome-scale protein function classification. *Bioinforma. Oxf. Engl.* **30**, 1236–1240 (2014).](https://www.zotero.org/google-docs/?QWD4qM)
31. [Mitchell, A. L., *et al.* InterPro in 2019: improving coverage, classification and access to protein sequence annotations. *Nucleic Acids Res.* **47**, D351–D360 (2018).](https://www.zotero.org/google-docs/?QWD4qM)
32. [Marchler-Bauer, A., *et al.* CDD/SPARCLE: functional classification of proteins via subfamily domain architectures. *Nucleic Acids Res.* **45**, D200–D203 (2016).](https://www.zotero.org/google-docs/?QWD4qM)
33. [Lewis, T. E., *et al.* Gene3D: Extensive prediction of globular domains in proteins. *Nucleic Acids Res.* **46**, D435–D439 (2017).](https://www.zotero.org/google-docs/?QWD4qM)
34. [Pedruzzi, I., *et al.* HAMAP in 2015: updates to the protein family classification and annotation system. *Nucleic Acids Res.* **43**, D1064–D1070 (2014).](https://www.zotero.org/google-docs/?QWD4qM)
35. [Piovesan, D., *et al.* MobiDB 3.0: more annotations for intrinsic disorder, conformational diversity and interactions in proteins. *Nucleic Acids Res.* **46**, D471–D476 (2017).](https://www.zotero.org/google-docs/?QWD4qM)
36. [Mi, H., Muruganujan, A., Ebert, D., Huang, X. & Thomas, P. D. PANTHER version 14: more genomes, a new PANTHER GO-slim and improvements in enrichment analysis tools. *Nucleic Acids Res.* **47**, D419–D426 (2018).](https://www.zotero.org/google-docs/?QWD4qM)
37. [Mi, H., *et al.* Protocol Update for large-scale genome and gene function analysis with the PANTHER classification system (v.14.0). *Nat. Protoc.* **14**, 703–721 (2019).](https://www.zotero.org/google-docs/?QWD4qM)
38. [El-Gebali, S., *et al.* The Pfam protein families database in 2019. *Nucleic Acids Res.* **47**, D427–D432 (2018).](https://www.zotero.org/google-docs/?QWD4qM)
39. [Käll, L., Krogh, A. & Sonnhammer, E. L. L. A Combined transmembrane topology and signal peptide prediction method. *J. Mol. Biol.* **338**, 1027–1036 (2004).](https://www.zotero.org/google-docs/?QWD4qM)
40. [Wu, C. H., *et al.* PIRSF: family classification system at the Protein Information Resource. *Nucleic Acids Res.* **32**, D112–D114 (2004).](https://www.zotero.org/google-docs/?QWD4qM)
41. Attwood, T. K., *et al.* The PRINTS database: a fine-grained protein sequence annotation and analysis resource—its status in 2012. Database [Internet] 2012. Available from: https://doi.org/10.1093/database/bas019 (2012).
42. [Bru, C., Courcelle, E., Carrère, S., Beausse, Y., Dalmar, S. & Kahn, D. The ProDom database of protein domain families: more emphasis on 3D. *Nucleic Acids Res.* **33**, D212–D215 (2005).](https://www.zotero.org/google-docs/?QWD4qM)
43. [Sigrist, C. J. A., *et al.* New and continuing developments at PROSITE. *Nucleic Acids Res.* **41**, D344–D347 (2012).](https://www.zotero.org/google-docs/?QWD4qM)
44. [Akiva, E., *et al.* The Structure–Function Linkage Database. *Nucleic Acids Res.* **42**, D521–D530 (2013).](https://www.zotero.org/google-docs/?QWD4qM)
45. [Letunic, I. & Bork, P. 20 years of the SMART protein domain annotation resource. *Nucleic Acids Res.* **46**, D493–D496 (2017).](https://www.zotero.org/google-docs/?QWD4qM)
46. [Wilson, D., *et al.* SUPERFAMILY—sophisticated comparative genomics, data mining, visualization and phylogeny. *Nucleic Acids Res.* **37**, D380–D386 (2008).](https://www.zotero.org/google-docs/?QWD4qM)
47. [Haft, D. H., Selengut, J. D. & White, O. The TIGRFAMs database of protein families. *Nucleic Acids Res.* **31**, 371–373 (2013).](https://www.zotero.org/google-docs/?QWD4qM)
48. [Fabregat, A., *et al.* 2017. The Reactome Pathway Knowledgebase. *Nucleic Acids Res.* **46**, D649–D655 (2017).](https://www.zotero.org/google-docs/?QWD4qM)
49. [Caspi, R., *et al.* The MetaCyc database of metabolic pathways and enzymes. *Nucleic Acids Res.* **46**, D633–D639 (2017).](https://www.zotero.org/google-docs/?QWD4qM)
50. Kanehisa, M. & Goto, S. KEGG: Kyoto Encyclopedia of Genes and Genomes. *Nucleic Acids Res.* **28**, 27-30 (2000).
51. Kanehisa, M. Toward understanding the origin and evolution of cellular organisms. *Protein Sci.* **28**, 1947-1951 (2019).
52. Kanehisa, M., Furumichi, M., Sato, Y., Ishiguro-Watanabe, M. & Tanabe, M. KEGG: integrating viruses and cellular organisms. *Nucleic Acids Res.* **49,** (2021) https://doi.org/10.1093/nar/gkaa970.
53. Nielsen, H. Predicting Secretory Proteins with SignalP. In: Kihara D, editor. Protein Function Prediction: Methods and Protocols. New York, NY: Springer New York. p. 59–73. Available from: https://doi.org/10.1007/978-1-4939-7015-5_6 (2017).
54. [Krogh, A., Larsson, B., von Heijne, G. & Sonnhammer, E. L. L. Predicting transmembrane protein topology with a hidden markov model: application to complete genomes. *J. Mol. Biol.* **305**, 567–580 (2001).](https://www.zotero.org/google-docs/?QWD4qM)
55. [Rahman, O., Cummings, S. P., Harrington, D. J. & Sutcliffe, I. C. Methods for the bioinformatic identification of bacterial lipoproteins encoded in the genomes of Gram-positive bacteria. *World J. Microbiol. Biotechnol.* **24**, 2377 (2008).](https://www.zotero.org/google-docs/?QWD4qM)
56. [Kanehisa, M., Sato, Y. & Morishima, K. BlastKOALA and GhostKOALA: KEGG Tools for Functional Characterization of Genome and Metagenome Sequences. *Comput. Resour. Mol. Biol.* **428**, 726–731 (2016).](https://www.zotero.org/google-docs/?QWD4qM)
57. [Blin, K., *et al.* AntiSMASH 4.0—improvements in chemistry prediction and gene cluster boundary identification. *Nucleic Acids Res.* **45**, W36–W41 (2017).](https://www.zotero.org/google-docs/?QWD4qM)
58. [Huerta-Cepas, J., *et al.* eggNOG 5.0: a hierarchical, functionally and phylogenetically annotated orthology resource based on 5090 organisms and 2502 viruses. *Nucleic Acids Res.* **47**, D309–D314 (2018).](https://www.zotero.org/google-docs/?QWD4qM)
59. [Buchfink, B., Xie, C. & Huson, D. H. Fast and sensitive protein alignment using DIAMOND. *Nat. Methods* **12**, 59 (2014).](https://www.zotero.org/google-docs/?QWD4qM)
60. [Aziz, R. K., *et al.* The RAST Server: Rapid annotations using subsystems technology. *BMC Genomics* **9**, 75 (2008).](https://www.zotero.org/google-docs/?QWD4qM)
61. [Overbeek, R., *et al.* The SEED and the Rapid Annotation of microbial genomes using Subsystems Technology (RAST). *Nucleic Acids Res.* **42**, D206–D214 (2013).](https://www.zotero.org/google-docs/?QWD4qM)
62. [Conesa, A., Götz, S., García-Gómez, J. M., Terol, J., Talón, M. & Robles, M. Blast2GO: a universal tool for annotation, visualization and analysis in functional genomics research. *Bioinformatics* **21**, 3674–3676 (2005).](https://www.zotero.org/google-docs/?QWD4qM)
63. [Galperin, M. Y., Makarova, K. S., Wolf, Y. I. & Koonin, E. V. 2015. Expanded microbial genome coverage and improved protein family annotation in the COG database. *Nucleic Acids Res.* **43**, D261–D269 (2015).](https://www.zotero.org/google-docs/?QWD4qM)
64. [Jia, B., *et al.* CARD 2017: expansion and model-centric curation of the comprehensive antibiotic resistance database. *Nucleic Acids Res.* **45**, D566–D573 (2017).](https://www.zotero.org/google-docs/?QWD4qM)
65. [Eswar, N., *et al.* Comparative protein structure modeling using modeller. *Curr. Protoc. Bioinforma.* **15**, 5.6.1-5.6.30 (2006).](https://www.zotero.org/google-docs/?QWD4qM)
66. Kahlon, R. S. Pseudomonas: Genome and Comparative Genomics. In: Kahlon RS, editor. Pseudomonas: Molecular and Applied Biology. Cham: Springer International Publishing. p. 127–191. Available from: https://doi.org/10.1007/978-3-319-31198-2_4 (2016).
67. [Recinos, D. A., *et al.* Redundant phenazine operons in Pseudomonas aeruginosa exhibit environment-dependent expression and differential roles in pathogenicity. *Proc. Natl. Acad. Sci. U. S. A.* **109**, 19420–19425 (2012).](https://www.zotero.org/google-docs/?QWD4qM)
68. [Eren AM, Esen ÖC, Quince C, Vineis JH, Morrison HG, Sogin ML, Delmont TO. 2015. Anvi’o: an advanced analysis and visualization platform for ‘omics data.van Gulik W, editor. PeerJ 3:e1319.](https://www.zotero.org/google-docs/?QWD4qM)
69. [Simão, F. A., Waterhouse, R. M., Ioannidis, P., Kriventseva, E. V. & Zdobnov, E. M. BUSCO: assessing genome assembly and annotation completeness with single-copy orthologs. *Bioinformatics* **31**, 3210–3212 (2015).](https://www.zotero.org/google-docs/?QWD4qM)
70. García-Valdés, E. & Lalucat, J. *Pseudomonas*: Molecular Phylogeny and Current Taxonomy. In: Kahlon RS, editor. *Pseudomonas*: Molecular and Applied Biology. Cham: *Springer International Publishing.* p. 1–23. Available from: https://doi.org/10.1007/978-3-319-31198-2_1 (2016).
71. [Mandalakis, M., *et al.* Microbial strains isolated from CO2-venting Kolumbo submarine volcano show enhanced co-tolerance to acidity and antibiotics. *Mar. Environ. Res.* **144**, 102–110 (2019).](https://www.zotero.org/google-docs/?QWD4qM)

**Supplementary Table 1.** Environmental characteristics (sampling depth, pH, Temp) and phenotypic traits to six antibiotics (Amp, Eryth, Cipr, Cef, Tetr, Chlr), four heavy metals (As, Sb, Sr, Hg) and acidity (pH tolerance) of the selected strains from Kolumbo volcano. Data were obtained by Mandalakis et al. 2019^71^. Amp: ampicillin; Eryth: erythromycin; Cipr: ciprofloxacin; Cef: cefuroxime; Tetr: tetracycline; Chlr: chloramphenicol; As: arsenic; Sb: antimony; Sr: strontium; Hg: mercury.

**Supplementary Table 2.** Busco search results of the 21 strains.

| **Strain Code** | **Busco Results** | | |
| --- | --- | --- | --- |
|  | *Missing OGs* | *Annotation of missing OGs* | *Duplicate OGs* |
| Strain05 Strain06 Strain07 Strain16 Strain19 Strain20 Strain21 Strain23 | POG09090294 | hypothetical protein | POG09090081, POG0909039L |
| Strain04 Strain11 Strain22 | POG090901TU | Biotin-acetyl-CoA-carboxylase ligase | POG0909039L |
| Strain02 Strain03 Strain12 Strain18 | none | - | POG09090233, POG0909039L |
| Strain01 | none | - | POG0909011A, POG09090233, POG090902NM, POG0909039L |
| Strain14 Strain24 | none | - | POG0909039L |
| Strain09 | POG090903GX | ribonuclease R | POG0909011K, POG09090233, POG090902NM, POG0909039L |
| Strain10 | none | - | POG0909011K, POG09090233, POG090902NM, POG0909039L |
| Strain08 | none | - | not listed (85 OGs in total) |

**Supplementary Table 3**. Identified Gene Clusters (GCs) of the Pangenome analysis.

| **Group** | **Number of Gene Clusters** | **Group Description** |
| --- | --- | --- |
| Gene Clusters Total | 10908 | All the Gene Clusters found by the Pangenome analysis of the Pseudomonas strains |
| Core | 2059 | GCs that can be found in all (21) strains |
| Aeruginosa unique | 2775 | GCs that can be found in all (8) Aeruginosa strains but not in other strains |
| Stutzeri unique | 643 | GCs that can be found in all (13) Stutzeri strains but not in other strains |
| Aeruginosa | 4834 | GCs that can be found in all (8) Aeruginosa strains. It is the sum of the groups Core + Aeruginosa unique |
| Stutzeri | 2702 | GCs that can be found in all (13) Stutzeri strains. It is the sum of the groups Core + Stutzeri unique |
| All Aeruginosa | 6530 | GCs that can be found in at least one Aeruginosa strain |
| All Stutzeri | 7190 | GCs that can be found in at least one Stutzeri strain |

**Supplementary Table 4.** Results of KEGG function^50-52^ analysis. A) List of KEGG functions in Core group. B) List of KEGG functions in Aeruginosa group. C) List of KEGG functions that are unique in Aeruginosa group. D) List of KEGG functions in Stutzeri group. C) List of KEGG functions that are unique in Stutzeri group.

1. KEGG Core functions

| **source** | **accession** | **function** | **Num_Core_GCs** |
| --- | --- | --- | --- |
| KEGG_Function | 2000 | Transporters [BR:ko02000] | 165 |
| KEGG_Function | 99980 | Enzymes with EC numbers | 72 |
| KEGG_Function | 99997 | Function unknown | 72 |
| KEGG_Function | 3400 | DNA repair and recombination proteins [BR:ko03400] | 71 |
| KEGG_Function | 2035 | Bacterial motility proteins [BR:ko02035] | 70 |
| KEGG_Function | 3016 | Transfer RNA biogenesis [BR:ko03016] | 70 |
| KEGG_Function | 3009 | Ribosome biogenesis [BR:ko03009] | 56 |
| KEGG_Function | 3011 | Ribosome [BR:ko03011] | 50 |
| KEGG_Function | 4147 | Exosome [BR:ko04147] | 39 |
| KEGG_Function | 3000 | Transcription factors [BR:ko03000] | 37 |
| KEGG_Function | 1002 | Peptidases [BR:ko01002] | 36 |
| KEGG_Function | 2022 | Two-component system [BR:ko02022] | 30 |
| KEGG_Function | 3110 | Chaperones and folding catalysts [BR:ko03110] | 29 |
| KEGG_Function | 3036 | Chromosome and associated proteins [BR:ko03036] | 28 |
| KEGG_Function | 1011 | Peptidoglycan biosynthesis and degradation proteins [BR:ko01011] | 27 |
| KEGG_Function | 99996 | General function prediction only | 26 |
| KEGG_Function | 3029 | Mitochondrial biogenesis [BR:ko03029] | 26 |
| KEGG_Function | 230 | Purine metabolism [PATH:ko00230] | 23 |
| KEGG_Function | 1005 | Lipopolysaccharide biosynthesis proteins [BR:ko01005] | 21 |
| KEGG_Function | 2044 | Secretion system [BR:ko02044] | 18 |
| KEGG_Function | 1004 | Lipid biosynthesis proteins [BR:ko01004] | 17 |
| KEGG_Function | 99982 | Energy metabolism | 16 |
| KEGG_Function | 99992 | Structural proteins | 16 |
| KEGG_Function | 240 | Pyrimidine metabolism [PATH:ko00240] | 15 |
| KEGG_Function | 760 | Nicotinate and nicotinamide metabolism [PATH:ko00760] | 15 |
| KEGG_Function | 99975 | Protein processing | 15 |
| KEGG_Function | 1007 | Amino acid related enzymes [BR:ko01007] | 15 |
| KEGG_Function | 860 | Porphyrin and chlorophyll metabolism [PATH:ko00860] | 14 |
| KEGG_Function | 770 | Pantothenate and CoA biosynthesis [PATH:ko00770] | 13 |
| KEGG_Function | 790 | Folate biosynthesis [PATH:ko00790] | 13 |
| KEGG_Function | 400 | Phenylalanine, tyrosine and tryptophan biosynthesis [PATH:ko00400] | 12 |
| KEGG_Function | 3012 | Translation factors [BR:ko03012] | 12 |
| KEGG_Function | 270 | Cysteine and methionine metabolism [PATH:ko00270] | 12 |
| KEGG_Function | 1504 | Antimicrobial resistance genes [BR:ko01504] | 12 |
| KEGG_Function | 630 | Glyoxylate and dicarboxylate metabolism [PATH:ko00630] | 12 |
| KEGG_Function | 3021 | Transcription machinery [BR:ko03021] | 12 |
| KEGG_Function | 99994 | Others | 10 |
| KEGG_Function | 2020 | Two-component system [PATH:ko02020] | 10 |
| KEGG_Function | 260 | Glycine, serine and threonine metabolism [PATH:ko00260] | 10 |
| KEGG_Function | 720 | Carbon fixation pathways in prokaryotes [PATH:ko00720] | 10 |
| KEGG_Function | 564 | Glycerophospholipid metabolism [PATH:ko00564] | 9 |
| KEGG_Function | 910 | Nitrogen metabolism [PATH:ko00910] | 9 |
| KEGG_Function | 520 | Amino sugar and nucleotide sugar metabolism [PATH:ko00520] | 9 |
| KEGG_Function | 340 | Histidine metabolism [PATH:ko00340] | 9 |
| KEGG_Function | 130 | Ubiquinone and other terpenoid-quinone biosynthesis [PATH:ko00130] | 9 |
| KEGG_Function | 3019 | Messenger RNA biogenesis [BR:ko03019] | 9 |
| KEGG_Function | 2025 | Biofilm formation - Pseudomonas aeruginosa [PATH:ko02025] | 9 |
| KEGG_Function | 280 | Valine, leucine and isoleucine degradation [PATH:ko00280] | 8 |
| KEGG_Function | 250 | Alanine, aspartate and glutamate metabolism [PATH:ko00250] | 8 |
| KEGG_Function | 4812 | Cytoskeleton proteins [BR:ko04812] | 8 |
| KEGG_Function | 4122 | Sulfur relay system [PATH:ko04122] | 8 |
| KEGG_Function | 670 | One carbon pool by folate [PATH:ko00670] | 8 |
| KEGG_Function | 680 | Methane metabolism [PATH:ko00680] | 8 |
| KEGG_Function | 330 | Arginine and proline metabolism [PATH:ko00330] | 8 |
| KEGG_Function | 2048 | Prokaryotic defense system [BR:ko02048] | 7 |
| KEGG_Function | 99995 | Signaling proteins | 7 |
| KEGG_Function | 900 | Terpenoid backbone biosynthesis [PATH:ko00900] | 7 |
| KEGG_Function | 194 | Photosynthesis proteins [BR:ko00194] | 7 |
| KEGG_Function | 620 | Pyruvate metabolism [PATH:ko00620] | 6 |
| KEGG_Function | 750 | Vitamin B6 metabolism [PATH:ko00750] | 6 |
| KEGG_Function | 220 | Arginine biosynthesis [PATH:ko00220] | 6 |
| KEGG_Function | 261 | Monobactam biosynthesis [PATH:ko00261] | 6 |
| KEGG_Function | 99976 | Replication and repair | 6 |
| KEGG_Function | 730 | Thiamine metabolism [PATH:ko00730] | 6 |
| KEGG_Function | 2024 | Quorum sensing [PATH:ko02024] | 5 |
| KEGG_Function | 300 | Lysine biosynthesis [PATH:ko00300] | 5 |
| KEGG_Function | 3032 | DNA replication proteins [BR:ko03032] | 5 |
| KEGG_Function | 190 | Oxidative phosphorylation [PATH:ko00190] | 5 |
| KEGG_Function | 710 | Carbon fixation in photosynthetic organisms [PATH:ko00710] | 4 |
| KEGG_Function | 780 | Biotin metabolism [PATH:ko00780] | 4 |
| KEGG_Function | 30 | Pentose phosphate pathway [PATH:ko00030] | 4 |
| KEGG_Function | 430 | Taurine and hypotaurine metabolism [PATH:ko00430] | 4 |
| KEGG_Function | 480 | Glutathione metabolism [PATH:ko00480] | 4 |
| KEGG_Function | 290 | Valine, leucine and isoleucine biosynthesis [PATH:ko00290] | 4 |
| KEGG_Function | 740 | Riboflavin metabolism [PATH:ko00740] | 4 |
| KEGG_Function | 61 | Fatty acid biosynthesis [PATH:ko00061] | 4 |
| KEGG_Function | 640 | Propanoate metabolism [PATH:ko00640] | 4 |
| KEGG_Function | 4146 | Peroxisome [PATH:ko04146] | 4 |
| KEGG_Function | 500 | Starch and sucrose metabolism [PATH:ko00500] | 4 |
| KEGG_Function | 1523 | Antifolate resistance [PATH:ko01523] | 4 |
| KEGG_Function | 362 | Benzoate degradation [PATH:ko00362] | 4 |
| KEGG_Function | 521 | Streptomycin biosynthesis [PATH:ko00521] | 3 |
| KEGG_Function | 99973 | Transcription | 3 |
| KEGG_Function | 930 | Caprolactam degradation [PATH:ko00930] | 3 |
| KEGG_Function | 99977 | Transport | 3 |
| KEGG_Function | 1006 | Prenyltransferases [BR:ko01006] | 3 |
| KEGG_Function | 920 | Sulfur metabolism [PATH:ko00920] | 3 |
| KEGG_Function | 4918 | Thyroid hormone synthesis [PATH:ko04918] | 3 |
| KEGG_Function | 99981 | Carbohydrate metabolism | 2 |
| KEGG_Function | 99985 | Amino acid metabolism | 2 |
| KEGG_Function | 99987 | Cofactor metabolism | 2 |
| KEGG_Function | 99978 | Cell growth | 2 |
| KEGG_Function | 4131 | Membrane trafficking [BR:ko04131] | 2 |
| KEGG_Function | 970 | Aminoacyl-tRNA biosynthesis [PATH:ko00970] | 2 |
| KEGG_Function | 791 | Atrazine degradation [PATH:ko00791] | 2 |
| KEGG_Function | 525 | Acarbose and validamycin biosynthesis [PATH:ko00525] | 2 |
| KEGG_Function | 1003 | Glycosyltransferases [BR:ko01003] | 2 |
| KEGG_Function | 4112 | Cell cycle - Caulobacter [PATH:ko04112] | 2 |
| KEGG_Function | 71 | Fatty acid degradation [PATH:ko00071] | 2 |
| KEGG_Function | 642 | Ethylbenzene degradation [PATH:ko00642] | 2 |
| KEGG_Function | 643 | Styrene degradation [PATH:ko00643] | 2 |
| KEGG_Function | 99979 | Unclassified viral proteins | 2 |
| KEGG_Function | 332 | Carbapenem biosynthesis [PATH:ko00332] | 2 |
| KEGG_Function | 99974 | Translation | 2 |
| KEGG_Function | 450 | Selenocompound metabolism [PATH:ko00450] | 2 |
| KEGG_Function | 1524 | Platinum drug resistance [PATH:ko01524] | 2 |
| KEGG_Function | 983 | Drug metabolism - other enzymes [PATH:ko00983] | 2 |
| KEGG_Function | 410 | beta-Alanine metabolism [PATH:ko00410] | 2 |
| KEGG_Function | 4932 | Non-alcoholic fatty liver disease (NAFLD) [PATH:ko04932] | 2 |
| KEGG_Function | 4070 | Phosphatidylinositol signaling system [PATH:ko04070] | 2 |
| KEGG_Function | 5134 | Legionellosis [PATH:ko05134] | 2 |
| KEGG_Function | 5120 | Epithelial cell signaling in Helicobacter pylori infection [PATH:ko05120] | 1 |
| KEGG_Function | 4213 | Longevity regulating pathway - multiple species [PATH:ko04213] | 1 |
| KEGG_Function | 5132 | Salmonella infection [PATH:ko05132] | 1 |
| KEGG_Function | 5206 | MicroRNAs in cancer [PATH:ko05206] | 1 |
| KEGG_Function | 99988 | Secondary metabolism | 1 |
| KEGG_Function | 72 | Synthesis and degradation of ketone bodies [PATH:ko00072] | 1 |
| KEGG_Function | 966 | Glucosinolate biosynthesis [PATH:ko00966] | 1 |
| KEGG_Function | 1009 | Protein phosphatases and associated proteins [BR:ko01009] | 1 |
| KEGG_Function | 1053 | Biosynthesis of siderophore group nonribosomal peptides [PATH:ko01053] | 1 |
| KEGG_Function | 1501 | beta-Lactam resistance [PATH:ko01501] | 1 |
| KEGG_Function | 2026 | Biofilm formation - Escherichia coli [PATH:ko02026] | 1 |
| KEGG_Function | 785 | Lipoic acid metabolism [PATH:ko00785] | 1 |
| KEGG_Function | 310 | Lysine degradation [PATH:ko00310] | 1 |
| KEGG_Function | 5016 | Huntington disease [PATH:ko05016] | 1 |
| KEGG_Function | 360 | Phenylalanine metabolism [PATH:ko00360] | 1 |
| KEGG_Function | 4934 | Cushing syndrome [PATH:ko04934] | 1 |
| KEGG_Function | 4931 | Insulin resistance [PATH:ko04931] | 1 |
| KEGG_Function | 460 | Cyanoamino acid metabolism [PATH:ko00460] | 1 |
| KEGG_Function | 99983 | Lipid metabolism | 1 |
| KEGG_Function | 1051 | Biosynthesis of ansamycins [PATH:ko01051] | 1 |
| KEGG_Function | 5014 | Amyotrophic lateral sclerosis (ALS) [PATH:ko05014] | 1 |
| KEGG_Function | 2042 | Bacterial toxins [BR:ko02042] | 1 |
| KEGG_Function | 650 | Butanoate metabolism [PATH:ko00650] | 1 |
| KEGG_Function | 440 | Phosphonate and phosphinate metabolism [PATH:ko00440] | 1 |
| KEGG_Function | 561 | Glycerolipid metabolism [PATH:ko00561] | 1 |
| KEGG_Function | 627 | Aminobenzoate degradation [PATH:ko00627] | 1 |
| KEGG_Function | 3320 | PPAR signaling pathway [PATH:ko03320] | 1 |
| KEGG_Function | 5204 | Chemical carcinogenesis [PATH:ko05204] | 1 |
| KEGG_Function | 5111 | Biofilm formation - Vibrio cholerae [PATH:ko05111] | 1 |
| KEGG_Function | 999 | Biosynthesis of secondary metabolites - unclassified [PATH:ko00999] | 1 |
| KEGG_Function | 982 | Drug metabolism - cytochrome P450 [PATH:ko00982] | 1 |
| KEGG_Function | 625 | Chloroalkane and chloroalkene degradation [PATH:ko00625] | 1 |
| KEGG_Function | 51 | Fructose and mannose metabolism [PATH:ko00051] | 1 |
| KEGG_Function | 524 | Neomycin, kanamycin and gentamicin biosynthesis [PATH:ko00524] | 1 |
| KEGG_Function | 380 | Tryptophan metabolism [PATH:ko00380] | 1 |

1. KEGG functions^50-52^ of Aeruginosa group

| **group** | **source** | **accession** | **function** | **Num_aeruginosa_GCs** |
| --- | --- | --- | --- | --- |
| aeruginosa | KEGG_Function | 2000 | Transporters [BR:ko02000] | 259 |
| aeruginosa | KEGG_Function | 2044 | Secretion system [BR:ko02044] | 93 |
| aeruginosa | KEGG_Function | 99997 | Function unknown | 83 |
| aeruginosa | KEGG_Function | 3000 | Transcription factors [BR:ko03000] | 50 |
| aeruginosa | KEGG_Function | 99980 | Enzymes with EC numbers | 49 |
| aeruginosa | KEGG_Function | 2035 | Bacterial motility proteins [BR:ko02035] | 47 |
| aeruginosa | KEGG_Function | 1504 | Antimicrobial resistance genes [BR:ko01504] | 30 |
| aeruginosa | KEGG_Function | 2022 | Two-component system [BR:ko02022] | 26 |
| aeruginosa | KEGG_Function | 2025 | Biofilm formation - Pseudomonas aeruginosa [PATH:ko02025] | 25 |
| aeruginosa | KEGG_Function | 1002 | Peptidases [BR:ko01002] | 20 |
| aeruginosa | KEGG_Function | 330 | Arginine and proline metabolism [PATH:ko00330] | 20 |
| aeruginosa | KEGG_Function | 99996 | General function prediction only | 18 |
| aeruginosa | KEGG_Function | 1004 | Lipid biosynthesis proteins [BR:ko01004] | 18 |
| aeruginosa | KEGG_Function | 3021 | Transcription machinery [BR:ko03021] | 18 |
| aeruginosa | KEGG_Function | 99995 | Signaling proteins | 17 |
| aeruginosa | KEGG_Function | 1005 | Lipopolysaccharide biosynthesis proteins [BR:ko01005] | 16 |
| aeruginosa | KEGG_Function | 860 | Porphyrin and chlorophyll metabolism [PATH:ko00860] | 15 |
| aeruginosa | KEGG_Function | 99977 | Transport | 14 |
| aeruginosa | KEGG_Function | 4147 | Exosome [BR:ko04147] | 14 |
| aeruginosa | KEGG_Function | 3400 | DNA repair and recombination proteins [BR:ko03400] | 12 |
| aeruginosa | KEGG_Function | 350 | Tyrosine metabolism [PATH:ko00350] | 11 |
| aeruginosa | KEGG_Function | 2024 | Quorum sensing [PATH:ko02024] | 9 |
| aeruginosa | KEGG_Function | 1007 | Amino acid related enzymes [BR:ko01007] | 9 |
| aeruginosa | KEGG_Function | 99994 | Others | 8 |
| aeruginosa | KEGG_Function | 2042 | Bacterial toxins [BR:ko02042] | 8 |
| aeruginosa | KEGG_Function | 564 | Glycerophospholipid metabolism [PATH:ko00564] | 7 |
| aeruginosa | KEGG_Function | 770 | Pantothenate and CoA biosynthesis [PATH:ko00770] | 7 |
| aeruginosa | KEGG_Function | 1011 | Peptidoglycan biosynthesis and degradation proteins [BR:ko01011] | 7 |
| aeruginosa | KEGG_Function | 340 | Histidine metabolism [PATH:ko00340] | 7 |
| aeruginosa | KEGG_Function | 2020 | Two-component system [PATH:ko02020] | 7 |
| aeruginosa | KEGG_Function | 627 | Aminobenzoate degradation [PATH:ko00627] | 7 |
| aeruginosa | KEGG_Function | 99992 | Structural proteins | 7 |
| aeruginosa | KEGG_Function | 3036 | Chromosome and associated proteins [BR:ko03036] | 7 |
| aeruginosa | KEGG_Function | 281 | Geraniol degradation [PATH:ko00281] | 6 |
| aeruginosa | KEGG_Function | 30 | Pentose phosphate pathway [PATH:ko00030] | 6 |
| aeruginosa | KEGG_Function | 1003 | Glycosyltransferases [BR:ko01003] | 6 |
| aeruginosa | KEGG_Function | 99982 | Energy metabolism | 6 |
| aeruginosa | KEGG_Function | 643 | Styrene degradation [PATH:ko00643] | 6 |
| aeruginosa | KEGG_Function | 99975 | Protein processing | 6 |
| aeruginosa | KEGG_Function | 190 | Oxidative phosphorylation [PATH:ko00190] | 6 |
| aeruginosa | KEGG_Function | 99986 | Glycan metabolism | 5 |
| aeruginosa | KEGG_Function | 1008 | Polyketide biosynthesis proteins [BR:ko01008] | 5 |
| aeruginosa | KEGG_Function | 280 | Valine, leucine and isoleucine degradation [PATH:ko00280] | 5 |
| aeruginosa | KEGG_Function | 930 | Caprolactam degradation [PATH:ko00930] | 5 |
| aeruginosa | KEGG_Function | 3110 | Chaperones and folding catalysts [BR:ko03110] | 5 |
| aeruginosa | KEGG_Function | 260 | Glycine, serine and threonine metabolism [PATH:ko00260] | 5 |
| aeruginosa | KEGG_Function | 3016 | Transfer RNA biogenesis [BR:ko03016] | 5 |
| aeruginosa | KEGG_Function | 910 | Nitrogen metabolism [PATH:ko00910] | 4 |
| aeruginosa | KEGG_Function | 480 | Glutathione metabolism [PATH:ko00480] | 4 |
| aeruginosa | KEGG_Function | 760 | Nicotinate and nicotinamide metabolism [PATH:ko00760] | 4 |
| aeruginosa | KEGG_Function | 3029 | Mitochondrial biogenesis [BR:ko03029] | 4 |
| aeruginosa | KEGG_Function | 920 | Sulfur metabolism [PATH:ko00920] | 4 |
| aeruginosa | KEGG_Function | 400 | Phenylalanine, tyrosine and tryptophan biosynthesis [PATH:ko00400] | 3 |
| aeruginosa | KEGG_Function | 270 | Cysteine and methionine metabolism [PATH:ko00270] | 3 |
| aeruginosa | KEGG_Function | 520 | Amino sugar and nucleotide sugar metabolism [PATH:ko00520] | 3 |
| aeruginosa | KEGG_Function | 620 | Pyruvate metabolism [PATH:ko00620] | 3 |
| aeruginosa | KEGG_Function | 3009 | Ribosome biogenesis [BR:ko03009] | 3 |
| aeruginosa | KEGG_Function | 3019 | Messenger RNA biogenesis [BR:ko03019] | 3 |
| aeruginosa | KEGG_Function | 650 | Butanoate metabolism [PATH:ko00650] | 3 |
| aeruginosa | KEGG_Function | 362 | Benzoate degradation [PATH:ko00362] | 3 |
| aeruginosa | KEGG_Function | 261 | Monobactam biosynthesis [PATH:ko00261] | 3 |
| aeruginosa | KEGG_Function | 3320 | PPAR signaling pathway [PATH:ko03320] | 3 |
| aeruginosa | KEGG_Function | 51 | Fructose and mannose metabolism [PATH:ko00051] | 3 |
| aeruginosa | KEGG_Function | 380 | Tryptophan metabolism [PATH:ko00380] | 3 |
| aeruginosa | KEGG_Function | 5230 | Central carbon metabolism in cancer [PATH:ko05230] | 2 |
| aeruginosa | KEGG_Function | 472 | D-Arginine and D-ornithine metabolism [PATH:ko00472] | 2 |
| aeruginosa | KEGG_Function | 536 | Glycosaminoglycan binding proteins [BR:ko00536] | 2 |
| aeruginosa | KEGG_Function | 361 | Chlorocyclohexane and chlorobenzene degradation [PATH:ko00361] | 2 |
| aeruginosa | KEGG_Function | 5418 | Fluid shear stress and atherosclerosis [PATH:ko05418] | 2 |
| aeruginosa | KEGG_Function | 2048 | Prokaryotic defense system [BR:ko02048] | 2 |
| aeruginosa | KEGG_Function | 99985 | Amino acid metabolism | 2 |
| aeruginosa | KEGG_Function | 250 | Alanine, aspartate and glutamate metabolism [PATH:ko00250] | 2 |
| aeruginosa | KEGG_Function | 99988 | Secondary metabolism | 2 |
| aeruginosa | KEGG_Function | 240 | Pyrimidine metabolism [PATH:ko00240] | 2 |
| aeruginosa | KEGG_Function | 99987 | Cofactor metabolism | 2 |
| aeruginosa | KEGG_Function | 1053 | Biosynthesis of siderophore group nonribosomal peptides [PATH:ko01053] | 2 |
| aeruginosa | KEGG_Function | 1501 | beta-Lactam resistance [PATH:ko01501] | 2 |
| aeruginosa | KEGG_Function | 430 | Taurine and hypotaurine metabolism [PATH:ko00430] | 2 |
| aeruginosa | KEGG_Function | 290 | Valine, leucine and isoleucine biosynthesis [PATH:ko00290] | 2 |
| aeruginosa | KEGG_Function | 740 | Riboflavin metabolism [PATH:ko00740] | 2 |
| aeruginosa | KEGG_Function | 360 | Phenylalanine metabolism [PATH:ko00360] | 2 |
| aeruginosa | KEGG_Function | 750 | Vitamin B6 metabolism [PATH:ko00750] | 2 |
| aeruginosa | KEGG_Function | 99993 | Cell motility | 2 |
| aeruginosa | KEGG_Function | 625 | Chloroalkane and chloroalkene degradation [PATH:ko00625] | 2 |
| aeruginosa | KEGG_Function | 790 | Folate biosynthesis [PATH:ko00790] | 2 |
| aeruginosa | KEGG_Function | 363 | Bisphenol degradation [PATH:ko00363] | 1 |
| aeruginosa | KEGG_Function | 960 | Tropane, piperidine and pyridine alkaloid biosynthesis [PATH:ko00960] | 1 |
| aeruginosa | KEGG_Function | 965 | Betalain biosynthesis [PATH:ko00965] | 1 |
| aeruginosa | KEGG_Function | 4071 | Sphingolipid signaling pathway [PATH:ko04071] | 1 |
| aeruginosa | KEGG_Function | 590 | Arachidonic acid metabolism [PATH:ko00590] | 1 |
| aeruginosa | KEGG_Function | 4216 | Ferroptosis [PATH:ko04216] | 1 |
| aeruginosa | KEGG_Function | 405 | Phenazine biosynthesis [PATH:ko00405] | 1 |
| aeruginosa | KEGG_Function | 600 | Sphingolipid metabolism [PATH:ko00600] | 1 |
| aeruginosa | KEGG_Function | 5034 | Alcoholism [PATH:ko05034] | 1 |
| aeruginosa | KEGG_Function | 633 | Nitrotoluene degradation [PATH:ko00633] | 1 |
| aeruginosa | KEGG_Function | 622 | Xylene degradation [PATH:ko00622] | 1 |
| aeruginosa | KEGG_Function | 537 | Glycosylphosphatidylinositol (GPI)-anchored proteins [BR:ko00537] | 1 |
| aeruginosa | KEGG_Function | 3012 | Translation factors [BR:ko03012] | 1 |
| aeruginosa | KEGG_Function | 10 | Glycolysis / Gluconeogenesis [PATH:ko00010] | 1 |
| aeruginosa | KEGG_Function | 4978 | Mineral absorption [PATH:ko04978] | 1 |
| aeruginosa | KEGG_Function | 130 | Ubiquinone and other terpenoid-quinone biosynthesis [PATH:ko00130] | 1 |
| aeruginosa | KEGG_Function | 5016 | Huntington disease [PATH:ko05016] | 1 |
| aeruginosa | KEGG_Function | 4112 | Cell cycle - Caulobacter [PATH:ko04112] | 1 |
| aeruginosa | KEGG_Function | 4934 | Cushing syndrome [PATH:ko04934] | 1 |
| aeruginosa | KEGG_Function | 630 | Glyoxylate and dicarboxylate metabolism [PATH:ko00630] | 1 |
| aeruginosa | KEGG_Function | 99983 | Lipid metabolism | 1 |
| aeruginosa | KEGG_Function | 670 | One carbon pool by folate [PATH:ko00670] | 1 |
| aeruginosa | KEGG_Function | 640 | Propanoate metabolism [PATH:ko00640] | 1 |
| aeruginosa | KEGG_Function | 4146 | Peroxisome [PATH:ko04146] | 1 |
| aeruginosa | KEGG_Function | 642 | Ethylbenzene degradation [PATH:ko00642] | 1 |
| aeruginosa | KEGG_Function | 440 | Phosphonate and phosphinate metabolism [PATH:ko00440] | 1 |
| aeruginosa | KEGG_Function | 500 | Starch and sucrose metabolism [PATH:ko00500] | 1 |
| aeruginosa | KEGG_Function | 3011 | Ribosome [BR:ko03011] | 1 |
| aeruginosa | KEGG_Function | 230 | Purine metabolism [PATH:ko00230] | 1 |
| aeruginosa | KEGG_Function | 220 | Arginine biosynthesis [PATH:ko00220] | 1 |
| aeruginosa | KEGG_Function | 194 | Photosynthesis proteins [BR:ko00194] | 1 |
| aeruginosa | KEGG_Function | 999 | Biosynthesis of secondary metabolites - unclassified [PATH:ko00999] | 1 |
| aeruginosa | KEGG_Function | 623 | Toluene degradation [PATH:ko00623] | 1 |
| aeruginosa | KEGG_Function | 5143 | African trypanosomiasis [PATH:ko05143] | 1 |
| aeruginosa | KEGG_Function | 410 | beta-Alanine metabolism [PATH:ko00410] | 1 |

1. KEGG Unique functions^50-52^ of Aeruginosa group

| **group** | **source** | **accession** | **function** | **Num_aeruginosa_GCs** |
| --- | --- | --- | --- | --- |
| aeruginosa | KEGG_Function | 281 | Geraniol degradation [PATH:ko00281] | 6 |
| aeruginosa | KEGG_Function | 5230 | Central carbon metabolism in cancer [PATH:ko05230] | 2 |
| aeruginosa | KEGG_Function | 472 | D-Arginine and D-ornithine metabolism [PATH:ko00472] | 2 |
| aeruginosa | KEGG_Function | 363 | Bisphenol degradation [PATH:ko00363] | 1 |
| aeruginosa | KEGG_Function | 960 | Tropane, piperidine and pyridine alkaloid biosynthesis [PATH:ko00960] | 1 |
| aeruginosa | KEGG_Function | 965 | Betalain biosynthesis [PATH:ko00965] | 1 |
| aeruginosa | KEGG_Function | 4071 | Sphingolipid signaling pathway [PATH:ko04071] | 1 |
| aeruginosa | KEGG_Function | 590 | Arachidonic acid metabolism [PATH:ko00590] | 1 |
| aeruginosa | KEGG_Function | 4216 | Ferroptosis [PATH:ko04216] | 1 |
| aeruginosa | KEGG_Function | 405 | Phenazine biosynthesis [PATH:ko00405] | 1 |
| aeruginosa | KEGG_Function | 600 | Sphingolipid metabolism [PATH:ko00600] | 1 |

1. KEGG functions^50-52^ of Stutzeri group

| **group** | **source** | **accession** | **function** | **Num_stutzeri_GCs** |
| --- | --- | --- | --- | --- |
| stutzeri | KEGG_Function | 2000 | Transporters [BR:ko02000] | 71 |
| stutzeri | KEGG_Function | 99997 | Function unknown | 23 |
| stutzeri | KEGG_Function | 2044 | Secretion system [BR:ko02044] | 15 |
| stutzeri | KEGG_Function | 2035 | Bacterial motility proteins [BR:ko02035] | 15 |
| stutzeri | KEGG_Function | 3400 | DNA repair and recombination proteins [BR:ko03400] | 11 |
| stutzeri | KEGG_Function | 99996 | General function prediction only | 10 |
| stutzeri | KEGG_Function | 99980 | Enzymes with EC numbers | 7 |
| stutzeri | KEGG_Function | 3000 | Transcription factors [BR:ko03000] | 6 |
| stutzeri | KEGG_Function | 3110 | Chaperones and folding catalysts [BR:ko03110] | 6 |
| stutzeri | KEGG_Function | 910 | Nitrogen metabolism [PATH:ko00910] | 5 |
| stutzeri | KEGG_Function | 1504 | Antimicrobial resistance genes [BR:ko01504] | 5 |
| stutzeri | KEGG_Function | 2020 | Two-component system [PATH:ko02020] | 5 |
| stutzeri | KEGG_Function | 3016 | Transfer RNA biogenesis [BR:ko03016] | 4 |
| stutzeri | KEGG_Function | 2022 | Two-component system [BR:ko02022] | 3 |
| stutzeri | KEGG_Function | 521 | Streptomycin biosynthesis [PATH:ko00521] | 3 |
| stutzeri | KEGG_Function | 99977 | Transport | 3 |
| stutzeri | KEGG_Function | 500 | Starch and sucrose metabolism [PATH:ko00500] | 3 |
| stutzeri | KEGG_Function | 3021 | Transcription machinery [BR:ko03021] | 3 |
| stutzeri | KEGG_Function | 3029 | Mitochondrial biogenesis [BR:ko03029] | 3 |
| stutzeri | KEGG_Function | 99975 | Protein processing | 3 |
| stutzeri | KEGG_Function | 260 | Glycine, serine and threonine metabolism [PATH:ko00260] | 3 |
| stutzeri | KEGG_Function | 1001 | Protein kinases [BR:ko01001] | 2 |
| stutzeri | KEGG_Function | 1011 | Peptidoglycan biosynthesis and degradation proteins [BR:ko01011] | 2 |
| stutzeri | KEGG_Function | 99995 | Signaling proteins | 2 |
| stutzeri | KEGG_Function | 99994 | Others | 2 |
| stutzeri | KEGG_Function | 30 | Pentose phosphate pathway [PATH:ko00030] | 2 |
| stutzeri | KEGG_Function | 290 | Valine, leucine and isoleucine biosynthesis [PATH:ko00290] | 2 |
| stutzeri | KEGG_Function | 99982 | Energy metabolism | 2 |
| stutzeri | KEGG_Function | 360 | Phenylalanine metabolism [PATH:ko00360] | 2 |
| stutzeri | KEGG_Function | 4147 | Exosome [BR:ko04147] | 2 |
| stutzeri | KEGG_Function | 99974 | Translation | 2 |
| stutzeri | KEGG_Function | 2025 | Biofilm formation - Pseudomonas aeruginosa [PATH:ko02025] | 2 |
| stutzeri | KEGG_Function | 190 | Oxidative phosphorylation [PATH:ko00190] | 2 |
| stutzeri | KEGG_Function | 3036 | Chromosome and associated proteins [BR:ko03036] | 2 |
| stutzeri | KEGG_Function | 562 | Inositol phosphate metabolism [PATH:ko00562] | 1 |
| stutzeri | KEGG_Function | 984 | Steroid degradation [PATH:ko00984] | 1 |
| stutzeri | KEGG_Function | 5020 | Prion diseases [PATH:ko05020] | 1 |
| stutzeri | KEGG_Function | 4973 | Carbohydrate digestion and absorption [PATH:ko04973] | 1 |
| stutzeri | KEGG_Function | 564 | Glycerophospholipid metabolism [PATH:ko00564] | 1 |
| stutzeri | KEGG_Function | 860 | Porphyrin and chlorophyll metabolism [PATH:ko00860] | 1 |
| stutzeri | KEGG_Function | 10 | Glycolysis / Gluconeogenesis [PATH:ko00010] | 1 |
| stutzeri | KEGG_Function | 99987 | Cofactor metabolism | 1 |
| stutzeri | KEGG_Function | 520 | Amino sugar and nucleotide sugar metabolism [PATH:ko00520] | 1 |
| stutzeri | KEGG_Function | 4978 | Mineral absorption [PATH:ko04978] | 1 |
| stutzeri | KEGG_Function | 99978 | Cell growth | 1 |
| stutzeri | KEGG_Function | 900 | Terpenoid backbone biosynthesis [PATH:ko00900] | 1 |
| stutzeri | KEGG_Function | 1003 | Glycosyltransferases [BR:ko01003] | 1 |
| stutzeri | KEGG_Function | 130 | Ubiquinone and other terpenoid-quinone biosynthesis [PATH:ko00130] | 1 |
| stutzeri | KEGG_Function | 3009 | Ribosome biogenesis [BR:ko03009] | 1 |
| stutzeri | KEGG_Function | 4112 | Cell cycle - Caulobacter [PATH:ko04112] | 1 |
| stutzeri | KEGG_Function | 630 | Glyoxylate and dicarboxylate metabolism [PATH:ko00630] | 1 |
| stutzeri | KEGG_Function | 1002 | Peptidases [BR:ko01002] | 1 |
| stutzeri | KEGG_Function | 4146 | Peroxisome [PATH:ko04146] | 1 |
| stutzeri | KEGG_Function | 230 | Purine metabolism [PATH:ko00230] | 1 |
| stutzeri | KEGG_Function | 362 | Benzoate degradation [PATH:ko00362] | 1 |
| stutzeri | KEGG_Function | 261 | Monobactam biosynthesis [PATH:ko00261] | 1 |
| stutzeri | KEGG_Function | 99992 | Structural proteins | 1 |
| stutzeri | KEGG_Function | 643 | Styrene degradation [PATH:ko00643] | 1 |
| stutzeri | KEGG_Function | 680 | Methane metabolism [PATH:ko00680] | 1 |
| stutzeri | KEGG_Function | 1007 | Amino acid related enzymes [BR:ko01007] | 1 |
| stutzeri | KEGG_Function | 330 | Arginine and proline metabolism [PATH:ko00330] | 1 |
| stutzeri | KEGG_Function | 5111 | Biofilm formation - Vibrio cholerae [PATH:ko05111] | 1 |
| stutzeri | KEGG_Function | 450 | Selenocompound metabolism [PATH:ko00450] | 1 |
| stutzeri | KEGG_Function | 1524 | Platinum drug resistance [PATH:ko01524] | 1 |
| stutzeri | KEGG_Function | 194 | Photosynthesis proteins [BR:ko00194] | 1 |
| stutzeri | KEGG_Function | 5143 | African trypanosomiasis [PATH:ko05143] | 1 |
| stutzeri | KEGG_Function | 920 | Sulfur metabolism [PATH:ko00920] | 1 |
| stutzeri | KEGG_Function | 983 | Drug metabolism - other enzymes [PATH:ko00983] | 1 |
| stutzeri | KEGG_Function | 4070 | Phosphatidylinositol signaling system [PATH:ko04070] | 1 |

1. KEGG Unique functions^50-52^ of Stutzeri group

| **group** | **source** | **accession** | **function** | **Num_stutzeri_GCs** |
| --- | --- | --- | --- | --- |
| stutzeri | KEGG_Function | 1001 | Protein kinases [BR:ko01001] | 2 |
| stutzeri | KEGG_Function | 562 | Inositol phosphate metabolism [PATH:ko00562] | 1 |
| stutzeri | KEGG_Function | 5020 | Prion diseases [PATH:ko05020] | 1 |
| stutzeri | KEGG_Function | 4973 | Carbohydrate digestion and absorption [PATH:ko04973] | 1 |
| stutzeri | KEGG_Function | 199 | Cytochrome P450 [BR:ko00199] | 0 |

**Supplementary Table 5.** List of phage-related GCs.

| **group** | **source** | **accession** | **function** | **Number of Gene Clusters** |
| --- | --- | --- | --- | --- |
| Core | ProDom | PD140749 | ORF PHAGE PA0727 | 1 |
| Core | TIGRFAM | TIGR02419 | C4_traR_proteo: phage/conjugal plasmid C-4 type zinc finger protein, TraR family | 1 |
| Core | Prokka | RefSeq:AAG04012.1 | putative bacteriophage protein | 1 |
|  |  |  |  |  |
| Aeruginosa | Prokka | RefSeq:AAG04111.1 | hypothetical protein of bacteriophage Pf1 | 2 |
| Aeruginosa | COG_FUNCTION | COG4385 | Bacteriophage P2-related tail formation | 1 |
| Aeruginosa | Interpro | IPR019289 | Bacteriophage tail protein Gp41, putative | 1 |
| Aeruginosa | Interpro | IPR012449 | Bacteriophage F116, Orf28 | 1 |
| Aeruginosa | PfamInterPro | PF16085 | Bacteriophage holin Hol, superfamily III | 1 |
| Aeruginosa | Rast_FigFam | fig\|287.7261.peg.5312 | Helix destabilizing protein of bacteriophage Pf1 | 1 |
| Aeruginosa | COG_FUNCTION | COG5004 | P2-like prophage tail protein X | 1 |
| Aeruginosa | COG_FUNCTION | COG3497 | Phage tail sheath protein FI | 1 |
| Aeruginosa | COG_FUNCTION | COG3498 | Phage tail tube protein FII | 1 |
| Aeruginosa | PfamInterPro | PF09684 | Phage tail protein (Tail_P2_I) | 1 |
| Aeruginosa | Interpro | IPR008861 | Phage Tail Protein X-like | 1 |
| Aeruginosa | PfamInterPro | PF10109 | Phage tail assembly chaperone proteins | 1 |
| Aeruginosa | PfamInterPro | PF12571 | Phage tail-collar fibre protein | 1 |
| Aeruginosa | Rast_FigFam | fig\|287.7261.peg.572 | FIG032563: Phage tail protein D | 1 |
| Aeruginosa | SUPERFAMILY | SSF69279 | Phage tail proteins | 1 |
| Aeruginosa | COG_FUNCTION | COG3628 | Phage baseplate assembly protein W | 1 |
| Aeruginosa | COG_FUNCTION | COG5283 | Phage-related tail protein | 1 |
| Aeruginosa | COG_FUNCTION | COG4540 | Phage P2 baseplate assembly protein gpV | 1 |
| Aeruginosa | COG_FUNCTION | COG3499 | Phage protein U | 1 |
| Aeruginosa | COG_FUNCTION | COG3948 | Phage-related baseplate assembly protein | 1 |
| Aeruginosa | COG_FUNCTION | COG3501 | Uncharacterized conserved protein, implicated in type VI secretion and phage assembly | 1 |
| Aeruginosa | EGGNOG | Dd1591_1833 | Phage conjugal plasmid C-4 type zinc finger protein, TraR family | 1 |
| Aeruginosa | Interpro | IPR032637 | Putative phage holin-like | 1 |
| Aeruginosa | Interpro | IPR008473 | Putative 3TM holin, Phage holin 3 | 1 |
| Aeruginosa | Prokka | RefSeq:BAN56475.1 | putative prophage P4 integrase | 1 |
| Aeruginosa | Rast_FigFam | fig\|287.7261.peg.2073 | Phage terminase, small subunit | 1 |
| Aeruginosa | SUPERFAMILY | SSF69349 | Phage fibre proteins | 1 |
| Aeruginosa | PfamInterPro | PF00589 | Phage integrase family | 2 |
| Aeruginosa | COG_FUNCTION | COG1842 | Phage shock protein A | 1 |
|  |  |  |  |  |
| Stutzeri | Prokka | RefSeq:AWT10893.1 | phage holin family protein | 1 |
|  |  |  |  |  |
|  |  |  | Total phage related GCs in Aeruginosa group | 34 |
|  |  |  | Total phage related GCs in Stutzeri group | 4 |

**Supplementary Table 6.** List of sigma (σ) factors.

| **group** | **source** | **function** | **Number of Gene Clusters** |
| --- | --- | --- | --- |
| core | EggnogKegg | rpoD; RNA polymerase primary sigma factor | 1 |
| core | EggnogKegg | fliA; RNA polymerase sigma factor for flagellar operon FliA | 1 |
| core | EggnogKegg | rpoH; RNA polymerase sigma-32 factor | 1 |
| core | EggnogKegg | rpoE; RNA polymerase sigma-70 factor, ECF subfamily | 1 |
| core | EggnogKegg | rpoS; RNA polymerase nonessential primary-like sigma factor | 1 |
| core | EggnogKegg | rpoN; RNA polymerase sigma-54 factor | 1 |
| core | EGGNOG | RNA polymerase sigma factor SigX | 1 |
|  |  |  |  |
| aeruginosa | EggnogKegg | rpoE; RNA polymerase sigma-70 factor, ECF subfamily | 11 |
| aeruginosa | EGGNOG | Inherit from bactNOG: RNA polymerase sigma-24 subunit, ECF subfamily | 1 |
| aeruginosa | EGGNOG | sigma factor | 1 |
| aeruginosa | KEGG_Genes | rpoE; RNA polymerase sigma-70 factor, ECF subfamily | 2 |
| aeruginosa | Prokka | Sigma factor PvdS, controling pyoverdin biosynthesis | 1 |
| aeruginosa | ProSitePatterns | Sigma-70 factors family signature 2. | 1 |
| aeruginosa | Rast_FigFam | Extracytoplasmic function (ECF) sigma factor VreI | 1 |
| aeruginosa | Rast_FigFam | Sigma-70 factor FpvI (ECF subfamily), controling pyoverdin biosynthesis | 1 |
|  |  |  |  |
| stutzeri | No matches for stuzeri |  |  |
|  |  | Total sigma factor-related GCs in Aeruginosa group | 26 |
|  |  | Total sigma factor-related GCs in Stutzeri group | 7 |

**Supplementary Table 7.** List of transposable elements.

| **group** | **source** | **accession** | **function** | **Number of Gene Clusters** |
| --- | --- | --- | --- | --- |
| Core | Blast2Go | P:GO:0006313 | P:transposition, DNA-mediated | 2 |
|  |  |  |  |  |
| Aeruginosa | COG_FUNCTION | COG3039 | Transposase and inactivated derivatives, IS5 family | 1 |
| Aeruginosa | COG_FUNCTION | COG3547 | Transposase | 1 |
|  |  |  |  |  |
| Stutzeri | no matches for stutzeri |  |  |  |
|  |  |  | Total transposase-related GCs in Aeruginosa group | 4 |
|  |  |  | Total transposase-related GCs in Stutzeri group | 2 |

**Supplementary Table 8.** List of GCs associated with antimicrobial and multidrug resistance of high and low tolerance strains.

| **group** | **source** | **function** | **Num_High_tolerance_GCs** |
| --- | --- | --- | --- |
| High_tolerance | KEGG_Function | Antimicrobial resistance genes [BR:ko01504] | 3 |
| High_tolerance | EggnogKegg | betI; TetR/AcrR family transcriptional regulator, transcriptional repressor of bet genes | 1 |
| High_tolerance | EggnogKegg | mexC; membrane fusion protein, multidrug efflux system | 1 |
| High_tolerance | EggnogKegg | mexD; multidrug efflux pump | 1 |
| High_tolerance | EggnogKegg | nfxB; TetR/AcrR family transcriptional regulator, mexCD-oprJ operon repressor | 1 |
| High_tolerance | TIGRFAM | 2A0602: RND transporter, hydrophobe/amphiphile efflux-1 (HAE1) family | 1 |
| High_tolerance | PRINTS | Acriflavin resistance protein family signature | 1 |
| High_tolerance | KEGG_Genes | betI; TetR/AcrR family transcriptional regulator, transcriptional repressor of bet genes | 1 |
| High_tolerance | Blast2Go | C:cell outer membrane; F:efflux transmembrane transporter activity; C:integral component of membrane; P:transmembrane transport | 1 |
| High_tolerance | Blast2Go | C:plasma membrane; P:drug transmembrane transport; F:efflux transmembrane transporter activity; C:integral component of membrane | 1 |
| High_tolerance | PANTHER_GO-Slim_Cellular | C:response to antibiotic; | 1 |
| High_tolerance | Interpo_GoTerms | F:efflux transmembrane transporter activity; C:membrane; F:transmembrane transporter activity; P:transmembrane transport | 1 |
| High_tolerance | KEGG_Genes | mexC; membrane fusion protein, multidrug efflux system | 1 |
| High_tolerance | KEGG_Genes | mexD; multidrug efflux pump | 1 |
| High_tolerance | Prokka | Multidrug efflux outer membrane protein OprJ precursor | 1 |
| High_tolerance | COG_FUNCTION | Multidrug efflux pump subunit AcrA (membrane-fusion protein) | 1 |
| High_tolerance | COG_FUNCTION | Multidrug efflux pump subunit AcrB | 1 |
| High_tolerance | Rast_FigFam | Multidrug efflux RND membrane fusion protein MexC | 1 |
| High_tolerance | Rast_FigFam | Multidrug efflux RND transporter MexD | 1 |
| High_tolerance | Gene3D | Multidrug efflux transporter AcrB TolC docking domain; DN and DC subdomains | 1 |
| High_tolerance | SUPERFAMILY | Multidrug efflux transporter AcrB transmembrane domain | 1 |
| High_tolerance | EGGNOG | Multidrug resistance protein | 1 |
| High_tolerance | KEGG_Genes | nfxB; TetR/AcrR family transcriptional regulator, mexCD-oprJ operon repressor | 1 |
| High_tolerance | KEGG_Genes | oprJ; outer membrane protein, multidrug efflux system | 1 |
| High_tolerance | PfamInterPro | Outer membrane efflux protein | 1 |
| High_tolerance | Gene3D | Outer membrane efflux proteins (OEP) | 1 |
| High_tolerance | SUPERFAMILY | Outer membrane efflux proteins (OEP) | 1 |
| High_tolerance | TIGRFAM | outer_NodT: efflux transporter, outer membrane factor (OMF) lipoprotein, NodT family | 1 |
| High_tolerance | Interpo_GoTerms | P:drug transmembrane transport; F:efflux transmembrane transporter activity; C:membrane; C:integral component of membrane; F:transmembrane transporter activity; P:transmembrane transport | 1 |
| High_tolerance | Prokka | Resistance-Nodulation-Cell Division (RND) multidrug efflux membrane fusion protein MexC precursor | 1 |
| High_tolerance | Prokka | Resistance-Nodulation-Cell Division (RND) multidrug efflux transporter MexD | 1 |
| High_tolerance | EGGNOG | RND efflux system, outer membrane lipoprotein | 1 |
| High_tolerance | Rast_FigFam | RND efflux system, outer membrane lipoprotein, NodT family | 1 |
| High_tolerance | TIGRFAM | RND_mfp: efflux transporter, RND family, MFP subunit | 1 |
| High_tolerance | EGGNOG | Transporter, hydrophobe amphiphile efflux-1 (HAE1) family | 1 |
|  |  |  |  |
| Low_tolerance | EGGNOG | glyoxalase bleomycin resistance protein dioxygenase | 1 |
| Low_tolerance | Interpro | Glyoxalase/Bleomycin resistance protein/Dihydroxybiphenyl dioxygenase | 1 |
| Low_tolerance | SUPERFAMILY | Glyoxalase/Bleomycin resistance protein/Dihydroxybiphenyl dioxygenase | 1 |

**Supplementary Table 9.** A list of tools databases and annotations that were used in the present study.

## FIGURES

**Supplementary Figure 1**

A) Statistics obtained from Quast analysis. Bars represent the N50 metric in Mb (left vertical axis) for the final output scaffolds and the initial Spades output scaffolds per strain. Lines represent the number of N’s per 100 kbp (right vertical axis) for the final output scaffolds and the initial Spades output scaffolds per strain. B) Bars represent the number of CDS found by prodigal per genome (left axis) and the line represents the percentage of these CDS that were annotated (right axis) in the final output. Red arrows indicate the strains of high tolerance to acidity, antibiotics and heavy metals.
